# Arf6-driven cell invasion is intrinsically linked to TRAK1-mediated mitochondrial anterograde trafficking to avoid oxidative catastrophe

**DOI:** 10.1038/s41467-018-05087-7

**Published:** 2018-07-11

**Authors:** Yasuhito Onodera, Jin-Min Nam, Mei Horikawa, Hiroki Shirato, Hisataka Sabe

**Affiliations:** 1 Department of Molecular Biology, Faculty of Medicine, Hokkaido University, 060-8638 Sapporo, Japan; 2 Global Institution for Collaborative Research and Education, Hokkaido University, 060-8638 Sapporo, Japan; 3 Department of Radiation Medicine, Faculty of Medicine, Hokkaido University, 060-8638 Sapporo, Japan

## Abstract

Mitochondria dynamically alter their subcellular localization during cell movement, although the underlying mechanisms remain largely elusive. The small GTPase Arf6 and its signaling pathway involving AMAP1 promote cell invasion via integrin recycling. Here we show that the Arf6–AMAP1 pathway promote the anterograde trafficking of mitochondria. Blocking the Arf6-based pathway causes mitochondrial aggregation near the microtubule-organizing center, and subsequently induces detrimental reactive oxygen species (ROS) production, likely via a mitochondrial ROS-induced ROS release-like mechanism. The Arf6-based pathway promotes the localization of ILK to focal adhesions to block RhoT1–TRAK2 association, which controls mitochondrial retrograde trafficking. Blockade of the RhoT1–TRAK1 machinery, rather than RhoT1–TRAK2, impairs cell invasion, but not two-dimensional random cell migration. Weakly or non-invasive cells do not notably express TRAK proteins, whereas they clearly express their mRNAs. Our results identified a novel association between cell movement and mitochondrial dynamics, which is specific to invasion and is necessary for avoiding detrimental ROS production.

## Abstract (web summary)

Mitochondria subcellular localization is dynamically regulated during migration. Here, the authors show that Arf6–AMAP1 dependent ILK localization at focal adhesions reduces mitochondrial retrograde trafficking in migratory cells and prevents mitochondrial aggregation and detrimental ROS production.

## Introduction

Cell movement is a complicated process, which requires the intracellular orchestration of numerous biochemical and cell-biological events. The dynamic relocation of mitochondria to particular subcellular sites has been observed in different types of cell movements; whereas mitochondria are concentrated at uropods during the chemotaxis of leukocytes^1^, mitochondrial redistribution towards leading edges is observed in the migration and/or invasion of fibroblasts and cancer cells^2–5^. Although the bioenergetic roles of mitochondria have been implicated to be crucial in cell movements^3,4^, the underlying mechanisms as to how mitochondrial dynamics is coordinated with cell movements, as well as biological implications of such mitochondrial relocation, still remain to be fully elucidated.

It is well documented that the increased production of reactive oxygen species (ROS), which is thought to be mainly via the mitochondrial respiratory chain, is closely associated with the malignant properties of cancer cells, including invasion and metastasis^6–8^. On the other hand, cancer cells also often show robust antioxidant capacity through the upregulation of antioxidant enzymes and the rewiring of cellular metabolism^7,8^. A number of anticancer treatments, including ionizing radiation (IR), directly or indirectly augment intracellular ROS production, which is shown to contribute to their anticancer effects^6,9^. Therefore, the high tolerance to ROS in cancer cells is thought to be intimately connected with their resistance to such therapies, and its modulation is considered a promising strategy for cancer treatment^6–9^.

The therapeutic resistance and invasiveness of cancer cells have often been observed concurrently and have thus been considered to be interconnected^10–12^. Integrins have predominant roles in the regulation of cell movements, including cancer invasion^13,14^, whereas they also facilitate resistance to therapies, including IR, through the activation of downstream signaling^13–16^. Although integrin-mediated signaling in cancers has been shown to promote their resistance to IR treatment^15–18^ as well as the enhancement of their invasiveness after IR^19–21^, involvement of the regulation of intracellular ROS levels in these contexts, possibly through the modulation of mitochondrial functions and/or positioning, has remained unknown.

We previously showed that the small GTPase Arf6 and its effector AMAP1, which are frequently overexpressed in cancers, have crucial roles in cancer invasion, metastasis, and also drug resistance^22–28^. Expression levels of Arf6 and AMAP1 are highly correlated with the invasive activities of cancer cells^26,27^, and these proteins promote the recycling back of internalized β1-integrins to the plasma membrane during cancer invasion. In this process, the Arf6–AMAP1 pathway uses protein kinase D2 (PRKD2), which directly binds to the cytoplasmic tail of β1-integrin^28,29^. Whereas the expression level of PRKD2 is not apparently changed in cancer cells and therefore is not the determinant of the formation of the Arf6–AMAP1–PRKD2 axis, the activation of Rab5c, another small GTPase, by epidermal growth factor receptor (EGFR) signaling acts as a positive regulator of the AMAP1–PRKD2 interaction^28,29^. Meanwhile, EGFR also activates Arf6 via the GTP-exchanging factor GEP100/BRAG2^23^, which is essential for the association of AMAP1 to Arf6 via its ArfGAP domain^27^. On the other hand, the Arf6–AMAP1 pathway may also contribute to drug resistance in the renal and breast cancer cells through as yet unidentified mechanisms^24,25^. Although the important roles of the Arf6–AMAP1–PRKD2 pathway in cancer invasion have been characterized as above, whether and/or how this pathway also affects cellular stress management, which would affect drug resistance, possibly through the modulation of integrin function and ROS regulation, are still largely unknown.

Here we show that the Arf6–AMAP1 pathway has pivotal roles in the control of mitochondrial positioning, which is crucial for the prevention of oxidative catastrophe as well as cell invasion, in highly invasive breast cancer cells. Blockade of this pathway increased intracellular ROS levels and induced mitochondrial aggregation near the microtubule-organizing center. ROS amplification was observed at dense networks of mitochondria caused by aggregation, which resembled the ROS-induced ROS release (RIRR) of cardiomyocytes^30–32^. The Arf6–AMAP1 pathway was required for the efficient localization of integrin-linked kinase (ILK) to focal adhesions (FAs), to regulate mitochondrial trafficking mediated by the motor adaptors RhoT and TRAK. RhoT-TRAK-mediated control of mitochondrial dynamics appeared to be more preferable in highly invasive breast cancer cells, and inhibition of anterograde mitochondrial trafficking impaired invasiveness and increased intracellular ROS levels. RIRR-like ROS amplification significantly enhanced cell death both in the steady state and after ROS induction by IR through the activation of caspase-3, whereas DNA damage and repair were not substantially affected. These findings indicate a novel molecular link between cell movements and mitochondrial dynamics, which appears to be crucial for both the invasive activity and tolerance to ROS of highly invasive cancers. Our findings may also lead to novel strategies to improve the efficacy of ROS-mediated cancer therapies, such as IR.

## Results

### Arf6–AMAP1 pathway regulates mitochondrial ROS levels

**Fig. 1:**
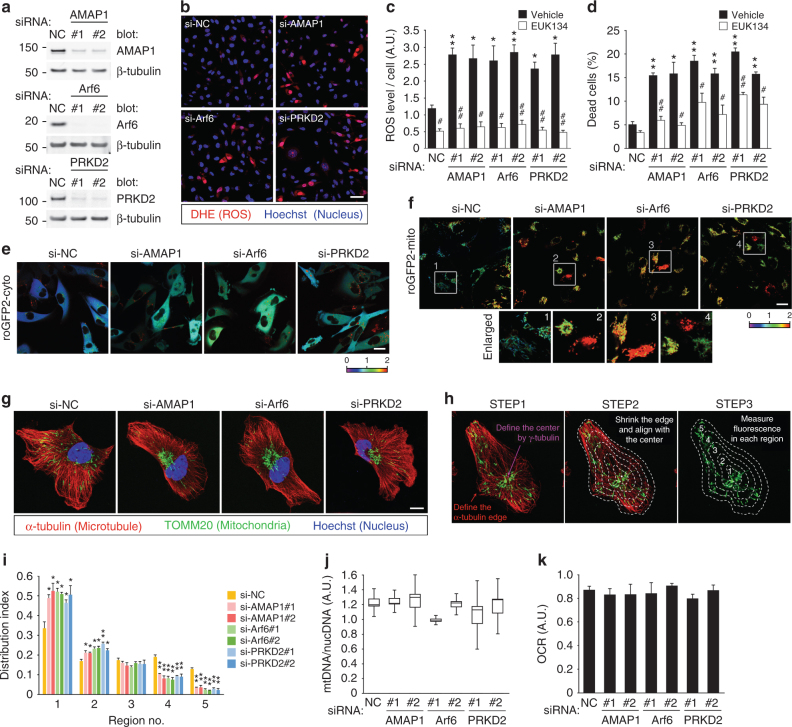
The Invasive machinery based on the Arf6–AMAP1 pathway regulates mitochondrial distribution and ROS levels. MDA-MB-231 cells were transfected with siRNAs (si-) targeting AMAP1, Arf6, or PRKD2, or the non-silencing control (NC) RNA duplex, as indicated. **a** Western blot of AMAP1, Arf6, or PRKD2. β-tubulin was used as a loading control (also for experiments hereafter). **b** ROS production was visualized by DHE (red). Nucleus staining with Hoechst 33342 (blue) is also shown. Bar, 50 μm. **c**, **d** Quantification of DHE fluorescence (**c**) and cumulative cell death (**d**) after siRNA transfection, in the presence or absence of 100 μM EUK-134. **e**, **f** Subcellular redox state analyzed by roGFP2 localized to the cytoplasm (cyto, **e**) or mitochondria (mito, **f**). The numbered white squares in (**f**) indicate the enlarged areas shown beneath. The fluorescence ratio of 405–488 nm excitation is shown by a heatmap. Bar, 20 μm. **g** Immunostaining of TOMM20 (green) and α-tubulin (red) by specific antibodies. The nucleus was stained with Hoechst 33342 (blue). Bar, 10 μm. **h** Scheme of the quantification of mitochondrial distribution. **i** Mitochondrial distribution indices quantified as described in Methods. **j** Ratio of mitochondrial to nuclear DNA copy numbers, measured as a function of mitochondrial biosynthesis. The graph is a box-and-whisker plot showing the first quartile, median, and third quartile, and the lower and upper error bars indicate the 1.5× interquartile range, respectively. **k** Relative oxygen consumption rate. All the graphs except for (**j**) indicate the mean ± standard error of the mean (SEM) for three independent experiments. * and ^#^*P* < 0.05; ** and ^##^*P* < 0.005 (two-tailed *t*-test, adjusted by the Holm–Sidak method). * and **, comparison to si-NC samples; ^#^ and ^##^, comparison to the corresponding vehicle-treated samples

To investigate possible roles of the Arf6–AMAP1 pathway in ROS regulation, we chose the highly invasive breast cancer cell line MDA-MB-231 as an experimental model, because we previously used these cells to analyze cancer invasion and integrin recycling mediated by this pathway^22,26–28^. siRNA-mediated blockade of the Arf6–AMAP1 pathway augmented ROS production and increased cell death in the steady state (Fig. 1a–d). The synthetic superoxide dismutase and catalase mimetic EUK-134^33^ substantially blocked the increase in ROS levels and cell death (Fig. 1c, d). To analyze spatial alterations in ROS levels, we utilized the genetically encoded redox sensor roGFP2^34^. Blockade of the Arf6–AMAP1 pathway clearly increased the oxidation of roGFP2 localized in the cytoplasm and mitochondria (Fig. 1e, f), whereas it affected roGFP2 oxidation to a lesser extent in nuclei and peroxisomes (Supplementary Fig. 1a, b).

We noticed that blockade of the Arf6–AMAP1 pathway substantially inhibited the cytoplasmic distribution of mitochondria (Fig. 1f). We thus quantified mitochondrial distribution over the microtubule network, and confirmed that the blockade of the Arf6–AMAP1 pathway indeed induced mitochondrial aggregation (Fig. 1g–i). The specificity of the above effect was estimated by analyzing the distribution of Rab7-positive vesicles, which correspond to late endosomes/lysosomes; whereas the knockdown of AMAP1 or PRKD2 did not notably affect the distribution of Rab7-positive vesicles, the knockdown of Arf6 clearly induced the aggregation of these vesicles (Supplementary Fig. 1c, d), suggesting that Arf6 might regulate the trafficking of late endosomes/lysosomes via other mechanisms. The copy number of mitochondrial DNA, a surrogate of mitochondrial biogenesis, and oxygen consumption rate (OCR) were largely unaffected (Fig. 1j, k). The phenotypes of siRNA-treated cells were largely rescued by the stable expression of corresponding cDNAs refractory to each siRNA (Supplementary Fig. 2a–f), further confirming that the above effects were not due to unexpected side effects. The increase in ROS was largely blocked by the expression of mitochondria-targeted catalase (mtCAT) and mitochondrial manganese superoxide dismutase (SOD2), whereas mitochondrial distribution was not restored (Supplementary Fig. 3a–d), suggesting that mitochondrial aggregation is a cause, rather than a consequence, of the increase in ROS production and cell death.

We previously reported that the regulation of β1-integrin recycling by the Arf6–AMAP1 pathway appears to be absent in weakly invasive breast cancer cells, such as MCF7^28^. Knockdown of AMAP1, Arf6, or PRKD2 did not induce mitochondrial aggregation nor increase ROS levels in MCF7 cells (Supplementary Fig. 4a–c). Steady-state cell death was slightly increased by PRKD2 knockdown (Supplementary Fig. 4d), probably due to other functions of this protein, which are not apparently associated with AMAP1 or Arf6 (as discussed below). AIIB2, a β1-integrin function blocking antibody, induced mitochondrial aggregation in MDA-MB-231 but not MCF7 cells (Supplementary Fig. 4e). These results further suggest that the above effects induced by inhibition of the Arf6–AMAP1–PRKD2 pathway were due to the inhibition of β1-integrin function, which resulted from impaired recycling.

### Mitochondrial aggregation amplifies intracellular ROS

**Fig. 2:**
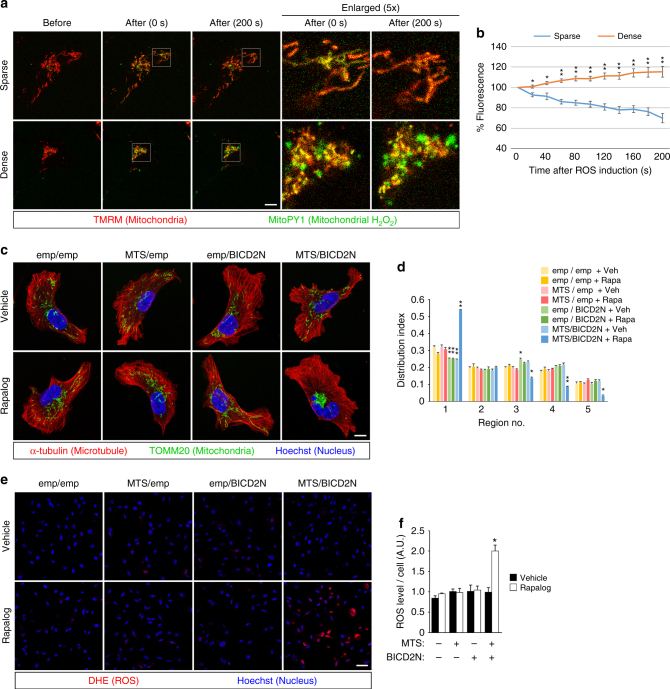
ROS Amplification by dense mitochondria within a single breast cancer cell. **a** Visualization of mitochondrial H_2_O_2_ in MDA-MB-231 cells. Mitochondria were labeled with TMRM (red), and H_2_O_2_ was visualized using MitoPY1 (green). Time-lapse images of the cells with sparse or dense mitochondria, before and after ROS induction by photoexcitation of TMRM, are shown. The white squares indicate the enlarged areas shown on the right. To avoid fluorescence saturation, the laser power for the excitation of TMRM (to visualize mitochondria) was reduced by several fold in dense condition. Bar, 10 μm. **b** Quantification of the time-dependent changes of MitoPY1 fluorescence in cells with dense or sparse mitochondrial networks. **c**–**f** MDA-MB-231 cells were stably transfected with FKBP-MTS (MTS) and/or BICD2N-FRB* (BICD2N), and treated with either vehicle or AP21967 (Rapalog). The empty vector (emp) was also used as a control. **c**, **d** Mitochondrial distribution was visualized by immunostaining (**c**), and quantified (**d**) as above. Bar, 10 μm. **e**, **f** ROS production was visualized by DHE (**e**), and quantified (**f**) as above. Bar, 50 μm. All graphs indicate the mean ± SEM for 10 (**b**) or three (**d**, **f**) independent experiments. * and ** indicate *P* < 0.05 and *P* < 0.005 (two-tailed *t*-test, adjusted by the Holm–Sidak method), compared to the corresponding samples (sparse vs. dense in **b**, emp/emp vs. others in **d** and **f**), respectively

A dense and aligned mitochondrial network in cardiomyocytes has been shown to amplify ROS via RIRR, in which the ROS leaking from damaged mitochondria further impairs adjacent mitochondria, leading to continuous ROS production and leakage as a chain reaction^30^. As ROS easily react with cytoplasmic molecules, the distance between each mitochondrion (i.e., mitochondrial density) is thought to be a key factor in the occurrence and outcome of RIRR^32^. We reasoned that mitochondrial aggregation caused by blockade of the Arf6-based pathway might also induce RIRR-like phenomena in single cancer cells. A ROS surge was induced by photoexcitation of tetramethylrhodamine methyl ester (TMRM), a mitochondria-targeted fluorescent dye, and ROS was monitored using MitoPY1, a mitochondria-targeted hydrogen peroxide (H_2_O_2_)-sensitive dye^35^. MitoPY1 fluorescence after ROS induction was cumulatively increased, whereas it was gradually decreased in cells with dense and sparse mitochondria, respectively (Fig. 2a, b). Likewise, after ROS induction in the cytoplasm by the photoinducible, genetically encoded ROS generator KillerRed^36^, MitoPY1 fluorescence increased significantly faster in mitochondria-dense areas compared with mitochondria-sparse areas (Supplementary Fig. 5a, b). These results indicate that intracellular ROS can indeed be further amplified by the dense mitochondrial network within a single cancer cell.

We also utilized a drug-regulatable intracellular trafficking system based on the rapamycin-binding domain of FKBP12 (FKBP) and the FKBP12-rapamycin-binding (FRB) domain of mammalian target of rapamycin (mTOR) harboring the T2098L mutation (FRB*)^37^. To control mitochondrial distribution, FKBP was anchored on the cytoplasmic surface of mitochondria using the membrane-targeting sequence (MTS) of *Listeria monocytogenes* ActA protein^38^, and FRB* was fused with the N-terminal region of Bicaudal-D2a (BICD2N), an adaptor/activator domain for dynein-dynactin. We confirmed that these fusion proteins were properly located in MDA-MB-231 cells (Supplementary Fig. 6a, b). Induction of heterodimerization between FKBP and FRB* by the rapamycin analog AP21967 (Rapalog), which specifically binds to mTOR-T2098L^39^, caused mitochondrial aggregation (Fig. 2c, d) and an increase in ROS levels (Fig. 2d, f), indicating that mitochondrial aggregation itself can indeed lead to an increase in ROS.

### RIRR-prone dense mitochondria results in ROS sensitization

**Fig. 3:**
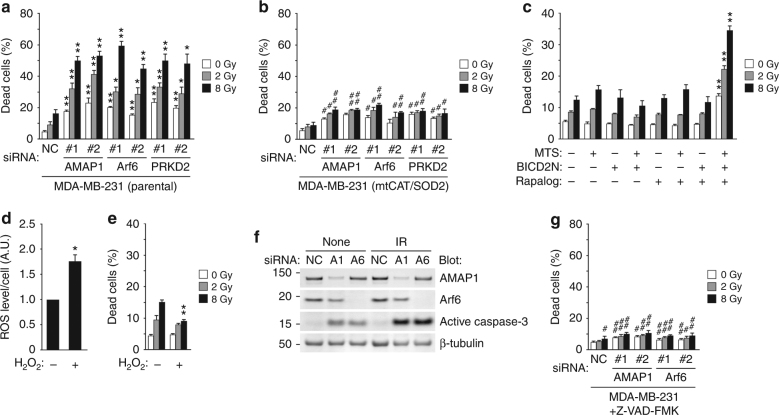
Mitochondrial aggregation enhances IR-induced cell death via amplification of ROS. **a**–**c** Cumulative cell death after irradiation of MDA-MB-231 cells: parental cells treated with siRNA targeting AMAP1, Arf6, or PRKD2 (**a**), cells stably transfected with mtCAT and SOD2 followed by the indicated siRNAs (**b**), and cells stably transfected with FKBP-MTS (MTS) and/or BICD2N-FRB* (BICD2N) followed by Rapalog treatment (**c**). **d**, **e** Quantification of DHE fluorescence before irradiation (**d**), and cumulative cell death after irradiation (**e**), in the presence or absence of 50 nM H_2_O_2_. **f** Activation of caspase-3 in MDA-MB-231 cells treated with the indicated siRNAs examined by western blot. **g** Cumulative cell death after irradiation of MDA-MB-231 cells with siRNA targeting AMAP1 or Arf6, in the presence of 50 μM Z-VAD-FMK. All graphs indicate the mean ± SEM of three independent experiments. * and ^#^*P* < 0.05; ** and ^##^*P* < 0.005 (two-tailed *t*-test, adjusted by the Holm–Sidak method). * and **, comparison to si-NC samples or non-treated cells; ^#^ and ^##^, comparison to the corresponding samples shown in (**a**)

Inhibition of β1-integrins is known to sensitize cancer cells to IR^15,17,18,21^, which generates ROS mainly by water radiolysis. Consistently, inhibition of the Arf6-based pathway induced significant radiosensitization (Fig. 3a). We reasoned that this radiosensitizing effect was due to ROS amplification by the dense mitochondria observed above. Indeed, the stable overexpression of mtCAT and SOD2 largely blocked, whereas drug-regulated mitochondrial aggregation induced radiosensitization (Fig. 3b, c). On the other hand, pretreatment with 50 nM H_2_O_2_, which appreciably increases intracellular ROS levels, did not increase (but rather decreased) cell death after irradiation (Fig. 3d, e), excluding the possibility that the increased ROS levels at the time of irradiation is crucial for radiosensitization.

Double-strand breaks (DSBs) of nuclear DNA, which are mediated by ROS, have been considered as the most relevant biological effect of IR^40^. Immunostaining of γ-H2AX, a well-established DSB marker, demonstrated that the numbers of γ-H2AX foci before and right after IR were not significantly changed by the knockdown of AMAP1, Arf6, or PRKD2 (Supplementary Fig. 7a, b). In addition, knockdown of AMAP1 or Arf6 did not affect the clearance of γ-H2AX foci observed 24 h after irradiation (Supplementary Fig. 7a, b). On the other hand, PRKD2 knockdown clearly delayed γ-H2AX foci clearance (Supplementary Fig. 7a, b). G2 arrest after IR, which occurs independently of p53^41^, is known to be essential for DNA damage repair^42,43^. Consistently, cell-cycle analysis using Fucci probes^44^ showed that the percentage of cells in the G1 phase was significantly increased by PRKD2 knockdown irrespective of IR treatment, whereas it was decreased by IR treatment under other conditions (Supplementary Fig. 7c). These results suggest that the inhibition of PRKD2 delays DSB repair, possibly by affecting the cell cycle, which does not appear to be relevant to the Arf6–AMAP1 pathway. To focus more closely on the Arf6–AMAP1–β1-integrin axis, we decided to exclude PRKD2 from our detailed analyses hereafter.

Despite the absence of a delay in DSB repair, knockdown of AMAP1 or Arf6 significantly induced the activation of caspase-3, which was further increased by IR (Fig. 3f). The pan-caspase inhibitor Z-VAD-FMK significantly reduced both steady state and IR-induced cell death augmented by blockade of the Arf6–AMAP1 pathway (Fig. 3g), collectively suggesting that mitochondrial ROS amplification, independent of nuclear DNA damage, leads to caspase-3 activation and induces cell death.

### ILK located at FAs regulates mitochondrial distribution

**Fig. 4:**
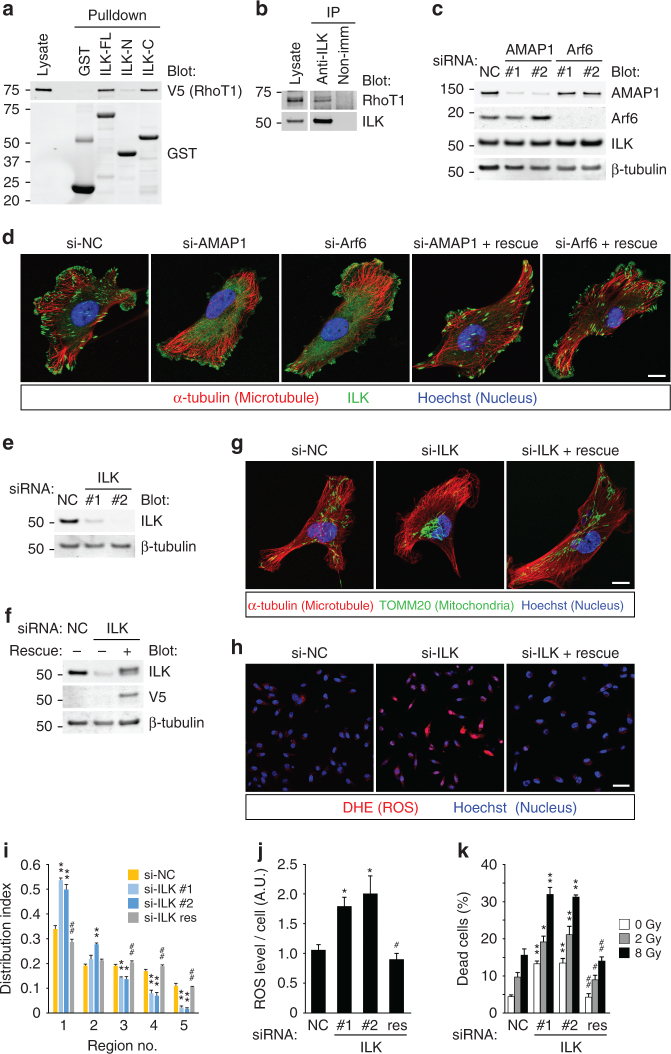
Localization of ILK at FAs is essential for regulation of mitochondrial distribution. **a** GST-pulldown assay using lysates from 293T cells overexpressing V5-tagged RhoT1 and the full length (ILK-FL), N terminus (ILK-N) or C terminus (ILK-C) of ILK fused to GST. GST alone (GST) was also used as a control. **b** Immunoprecipitation using MDA-MB-231 cell lysates and the anti-ILK antibody. Nonimmune rabbit IgG (non-imm) was also used as a control. **c**, **d** MDA-MB-231 cells were transfected with siRNAs targeting AMAP1 or Arf6. Protein expression of AMAP1, Arf6, or ILK was analyzed by western blot (**c**). ILK (green), microtubules (red), and nuclei (blue) were fluorescently visualized by specific antibodies or dyes. Bar, 10 μm (**d**). **e**–**k** MDA-MB-231 cells, either parental or stably transfected with ILK cDNA refractory to siRNA, were transfected with siRNAs targeting ILK. Protein expression of ILK was analyzed by western blot (**e**, **f**). Mitochondria (green), microtubules (red), and nuclei (blue) were fluorescently visualized by specific antibodies or dyes. Bar, 10 μm (**g**). Mitochondrial distribution indices were quantified (**i**). ROS production was visualized by DHE (**h**), and quantified (**j**). Bar, 50 μm. Cells were exposed to the indicated doses of IR, and cumulative cell death was measured (**k**). All graphs indicate the mean ± SEM for three independent experiments. * and ** indicate *P* < 0.05 and *P* < 0.005 (two-tailed *t*-test, adjusted by the Holm–Sidak method), compared to the corresponding samples (si-NC vs. si-ILK #1 or #2), respectively

Mitochondrial trafficking via microtubule motors is mediated by the interaction between several adaptor protein families, including RhoT/Miro^45,46^. Of note, proteome analyses suggested that RhoT1 and RhoT2 bind to ILK^47^, which is an integral component of integrin-mediated signaling^16,48^. We confirmed that ILK indeed binds to RhoT1 via its C-terminal portion containing a kinase domain (Fig. 4a), and endogenous proteins were coprecipitated by the immunoprecipitation of ILK (Fig. 4b). Knockdown of AMAP1 or Arf6 significantly impaired the localization of ILK at FAs, where it has central roles to regulate downstream signaling^16,48^, without affecting its protein levels (Fig. 4c, d). Despite the additional role mentioned above, PRKD2 and Rab5c were also essential for ILK localization at FAs (Supplementary Fig. 8 and data not shown), suggesting that the effect on ILK localization is indeed due to the inhibition of β1-integrin recycling. The phenotype was confirmed to be rescued by the expression of refractory cDNAs (Fig. 4d and Supplementary Fig. 8). ILK knockdown induced mitochondrial aggregation (Fig. 4e, g, i) and increased ROS levels and cell death both in the steady state and after IR (Fig. 4h, j, k). The phenotypes induced by ILK knockdown were largely rescued by the expression of a refractory cDNA of ILK (Fig. 4f–k). These data collectively suggest that ILK has fundamental roles in the Arf6–AMAP1–β1-integrin axis to regulate mitochondrial distribution and intracellular ROS levels.

**Fig. 5:**
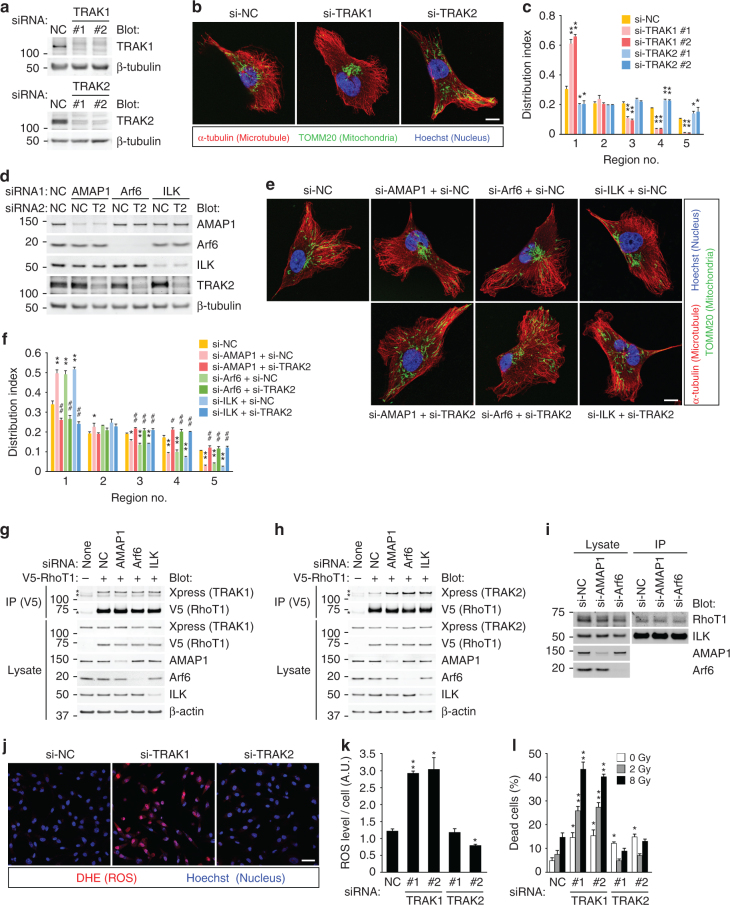
Arf6–AMAP1 pathway via ILK negatively regulates RhoT1–TRAK2 interaction for efficient distribution of mitochondria. **a**–**c** MDA-MB-231 cells were transfected with siRNA targeting TRAK1 or TRAK2. Protein expression of TRAK1 or TRAK2 was analyzed by western blotting (**a**). Mitochondria (green), microtubules (red), and nuclei (blue) were fluorescently visualized by specific antibodies or dyes. Bar, 10 μm (**b**). Mitochondrial distribution was quantified (**c**). **d**–**f** MDA-MB-231 cells were transfected with siRNA targeting AMAP1, Arf6, ILK, TRAK1, or TRAK2, in the combinations indicated. Expression of each protein was analyzed by western blotting (**d**). Mitochondria (green), microtubules (red), and nuclei (blue) were fluorescently visualized by specific antibodies or dyes. Bar, 10 μm (**e**). Mitochondrial distribution was quantified (**f**). **g**, **h** MDA-MB-231 cells stably expressing V5-RhoT1 were serially transfected with the indicated siRNAs and then plasmid DNA encoding Xpress-tagged TRAK1 or TRAK2. Parental cells were also used as a control. Immunoprecipitation by the anti-V5 antibody was performed using lysates of the cells expressing Xpress-TRAK1 (**g**) or TRAK2 (**h**). Asterisks indicate non-specific bands. (**i**) Immunoprecipitation using lysates of MDA-MB-231 cells transfected with the indicated siRNAs, and the anti-ILK antibody. **j**–**l** MDA-MB-231 cells were transfected with siRNA targeting TRAK1 or TRAK2. ROS production was visualized by DHE (**j**), and quantified (**k**). Bar, 50 μm. Cumulative cell death after IR treatment was also measured (**l**). All graphs indicate the mean ± SEM of three independent experiments. * and ^#^*P* < 0.05; ** and ^##^*P* < 0.005 (two-tailed *t*-test, adjusted by the Holm–Sidak method). * and **, comparison to si-NC samples; ^#^ and ^##^, comparison to the corresponding samples (i.e., si-AMAP1 + si-NC vs. si-AMAP1 + si-TRAK2)

RhoT is linked to microtubule motors, such as kinesin and dynein/dynactin, via another family of adaptor proteins, namely TRAK/Milton^37,45^. Previous studies suggested that TRAK1 associates with both kinesin and dynein/dynactin, whereas TRAK2 preferentially associates with dynein/dynactin, and such a difference might be attributed to different conformational regulations^37^. In MDA-MB-231 cells, TRAK1 knockdown inhibited, whereas TRAK2 knockdown promoted mitochondrial distribution (Fig. 5a–c), showing that their preferential roles in breast cancer cells are anterograde and retrograde trafficking of mitochondria, respectively. Moreover, TRAK2 knockdown almost completely canceled the mitochondrial aggregation, as well as the increase in ROS levels, induced by the knockdown of AMAP1, Arf6, or ILK (Fig. 5d–f and Supplementary Fig. 9a, b), indicating that TRAK2-mediated retrograde trafficking is upregulated when the Arf6–AMAP1–ILK axis is inhibited. Indeed, the association of RhoT1 to TRAK2, but not TRAK1, was appreciably increased by knockdown of AMAP1, Arf6, or ILK (Fig. 5g, h). On the other hand, the association of ILK and RhoT1 was not affected in these conditions (Fig. 5i), suggesting that formation of the ILK–RhoT1 complex occurs independently of the localization of ILK at FAs. We then visualized the intracellular distribution of the ILK–RhoT1 interaction by the proximity ligation assay (PLA)^49^. ILK-positive FAs and mitochondria were also visualized by stably expressed mEmerald-tagged ILK and additional staining using the TOMM20 antibody, respectively (Supplementary Fig. 10a, b). Notably, PLA signal spots were frequently observed at ILK-positive FAs, irrespective of the colocalization of mitochondria (Supplementary Fig. 10c). These results collectively suggest that RhoT1, which is not (yet) anchored to the mitochondrial surface, associates with cytoplasmic ILK, and is recruited to FAs. In accordance with the above results, knockdown of TRAK1, but not TRAK2, significantly increased intracellular ROS levels (Fig. 5j, k). TRAK1 knockdown also increased cell death in the steady state and after IR (Fig. 5l). On the other hand, TRAK2 knockdown increased cell death in the steady state, but not after IR, seemingly through mechanisms not related to ROS (Fig. 5l).

### TRAK1 promotes cancer invasion via mitochondrial trafficking

**Fig. 6:**
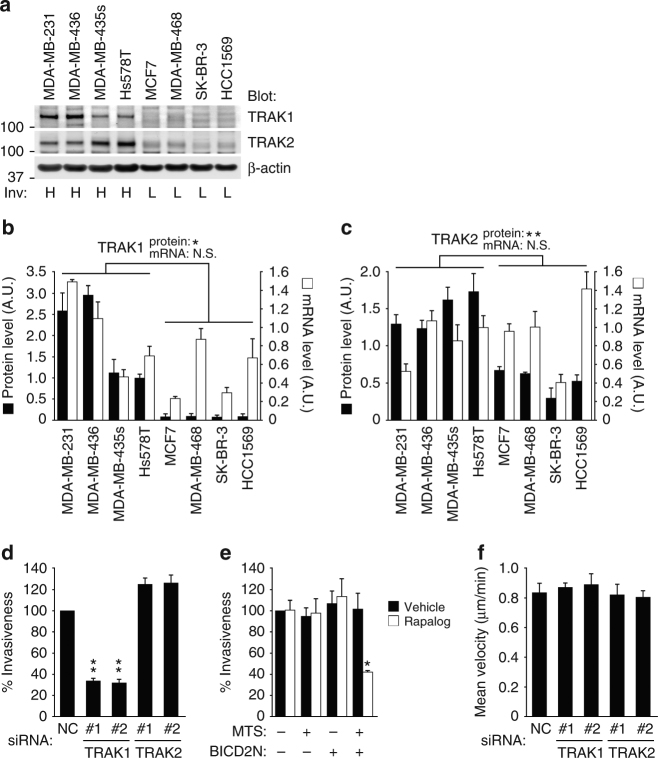
Regulation of mitochondrial trafficking via FAs is essential for cancer invasion. **a** Protein expression of TRAK1 and TRAK2 in different breast cancer cell lines analyzed by western blotting. β-actin was also analyzed as a control. Invasiveness (Inv) is indicated as either high (H) or low (L). **b**, **c** Protein (black bars) or mRNA (white bars) expression of TRAK1 (**b**) and TRAK2 (**c**) quantified by densitometry of the western blots (**a**) or qPCR, respectively. **d**, **e** Matrigel invasion of MDA-MB-231 cells transfected with siRNAs targeting TRAK1 or TRAK2 (**d**) or stably expressing FKBP-MTS (MTS) and/or BICD2N-FRB* (BICD2N) (**e**). In **e**, cells were treated with vehicle or AP21967 (Rapalog) before and during the assay, as indicated. **f** Velocity of the random migration of MDA-MB-231 cells transfected with siRNAs targeting TRAK1 or TRAK2. All the graphs indicate the mean ± SEM for three independent experiments. * and **P < 0.05 and *P* < 0.005 (two-tailed *t*-test, adjusted by the Holm–Sidak method), compared to the corresponding samples, respectively. N.S. not statistically significant

The above results suggest that mitochondrial positioning is under the control of FAs, which have fundamental roles in the context of cell movement^50–52^. We hypothesized that motile and invasive cells link the regulation of mitochondrial distribution and cell movement via the above-mentioned molecular mechanism. Protein expression levels of TRAK1 and TRAK2 were significantly high in breast cancer cells with high (H) invasiveness, compared to those with low (L) invasiveness (Fig. 6a–c). However, quantitative PCR (qPCR) analysis showed that TRAK1 and TRAK2 mRNA levels do not correlate with their protein levels, and are not significantly different between each group (Fig. 6b, c). Knockdown of TRAK1, but not TRAK2, clearly reduced the invasiveness of MD-MB-231 cells, even taking into account the effects on viability (Fig. 6d). Mitochondrial aggregation induced by the drug-regulatable heterodimerization system (Fig. 2) also reduced the invasiveness (Fig. 6e), suggesting that the peripheral distribution of mitochondria regulated by TRAKs is indeed favorable for cancer invasion. On the other hand, knockdown of TRAK1 or TRAK2 did not significantly affect the velocity of random cell migration on a collagen I-coated surface (Fig. 6f), suggesting that TRAK1-mediated anterograde trafficking is preferentially used for cell invasion.

**Fig. 7:**
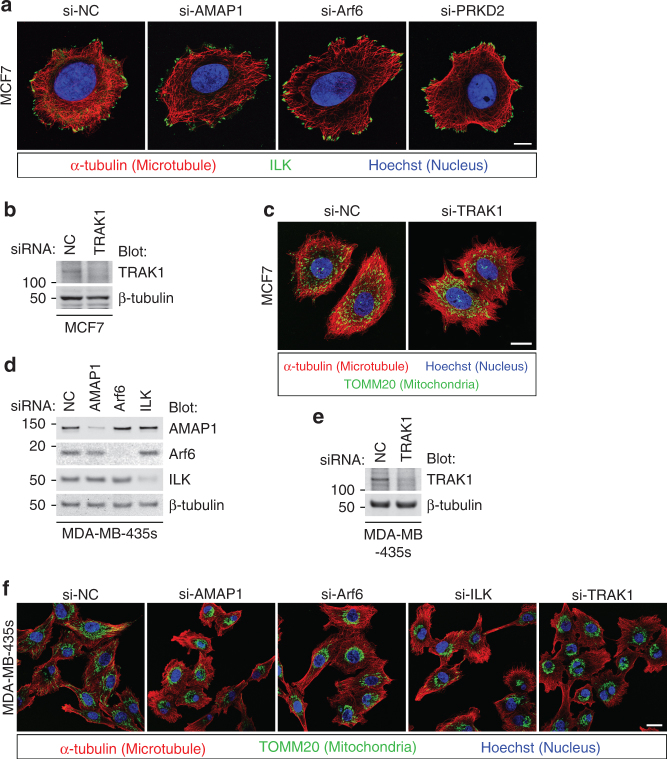
Utilization of the ILK–RhoT–TRAK axis for regulation of mitochondrial distribution in highly invasive breast cancer cells. **a**–**c** MCF7 cells were transfected with siRNAs targeting AMAP1, Arf6, PRKD2, or TRAK1. ILK (green), microtubules (red), and nuclei (blue) were fluorescently visualized by specific antibodies or dyes. Bar, 10 μm (**a**). Protein expression of TRAK1 was analyzed by western blotting (**b**). Mitochondria (green), microtubules (red), and nuclei (blue) were fluorescently visualized by specific antibodies or dyes. Bar, 20 μm (**c**). **d**–**f** MDA-MB-435s cells were transfected with siRNAs targeting AMAP1, Arf6, ILK, or TRAK1. Protein expression of AMAP1, Arf6, and ILK was analyzed by western blotting (**d**). TRAK1 expression was also examined separately (**e**). Mitochondria (green), microtubules (red), and nuclei (blue) were fluorescently visualized by specific antibodies or dyes. Bar, 20 μm (**f**)

Despite the marginal expression of TRAK1 and TRAK2 proteins, MCF7 cells showed a uniform distribution of mitochondria throughout the cytoplasm (Supplementary Fig. 3). Irrespective of the blockade of the Arf6–AMAP1–PRKD2 pathway, ILK localization was predominantly observed at peripheral punctate structures, which resembles AMAP1- or Arf6-deficient MDA-MB-231 cells (Fig. 7a, see also Fig. 4d). As MDA-MB-231 cells showed massive remodeling of deposited extracellular matrices, such as fibronectin (not shown), which is a characteristic of matured integrin-based adhesion structures^50^, MCF7 cells and siRNA-treated MDA-MB-231 cells hardly show such properties, suggesting that the adhesion structures in the latter cells remain less mature. Knockdown of TRAK1 did not affect the distribution of mitochondria in MCF7 cells (Fig. 7b, c), suggesting that some other adaptor proteins or TRAK2, despite its very low expression (Fig. 6a), might mediate mitochondrial distribution under the control of some mechanisms not associated with β1-integrin and ILK. On the other hand, in MDA-MB-435 s, which is another invasive breast cancer cell line, the knockdown of AMAP1, Arf6, ILK, or TRAK1 significantly inhibited mitochondrial distribution (Fig. 7d–f), suggesting that this molecular axis is indeed commonly used in highly invasive cells for regulating mitochondrial distribution.

## Discussion

The intracellular positioning of organelles, as well as their activities, are crucial for numerous cellular functions, including cell migration and invasion^2–5,53^. A number of studies have shown that the roles of mitochondria in bioenergetic and signaling are tightly associated with their localization^53^. In the present study, we showed that highly dense mitochondrial networks caused by an impairment of anterograde trafficking may lead to detrimental ROS amplification in single cells, highlighting the relocation of mitochondria as a potential regulatory mechanism for intracellular antioxidative capacity.

Previous studies have shown that the fragmentation and aggregation of mitochondria are observed after the induction of apoptotic cell death, although the roles of mitochondrial aggregation remain largely unknown^54,55^. Our findings suggest that mitochondrial aggregation on its own, which is mediated by motor-driven trafficking, can be a trigger, rather than a result of caspase-mediated cell death through detrimental ROS amplification (Fig. 3). On the other hand, apoptosis-induced mitochondrial aggregation was observed even in purified mitochondria^55^, suggesting that some alterations in properties of the mitochondrial outer membrane upon apoptosis promote mitochondrial aggregation. Nevertheless, such apoptosis-induced mitochondrial aggregation may also be followed by the ROS amplification via the RIRR-like mechanism observed in our study, which would further facilitate cell death. Although the cell death triggered by RIRR-like ROS amplification appears to be mediated by caspase-3 activation (Fig. 3), determining the type of cell death in this context requires additional investigation, which should be conducted in future studies.

RIRR in cardiomyocytes has been shown to be dependent on the mitochondrial permeability transition pore and inner membrane anion channel^30,31^. However, as mitochondria in cancer cells often demonstrate altered permeability^56^, the RIRR-like ROS amplification observed in breast cancer cells may be mediated by different mechanisms to those in cardiomyocytes. The increase in mitochondrial ROS production, which is often observed incancers and thought to promote some of their malignant properties^7–9^, may facilitate this mode of ROS amplification. Future studies are required to clarify whether a similar phenomenon can be observed, and if so, how it is prevented, in normal cells. In addition, the RIRR-like phenomenon observed in cancer cells occurred around the microtubule-organizing center (MTOC), where other ROS-generating organelles, such as the endoplasmic reticulum and peroxisomes might also be concentrated, leaving open the possibility that the interaction between mitochondria and such organelles is also involved in ROS amplification.

Integrin-mediated adhesion and signaling have pivotal roles in both cancer cells and normal cells. Among a number of signaling mediators that act downstream of integrins, FAK, Akt, NFκB, and their regulators have been well-characterized as important players for cell survival^13,14,16^. The mode of action of such integrin-mediated “survival signaling” known so far is largely through the modulation of signaling and/or transcriptional events that counteract death-inducing pathways^16,57,58^. On the other hand, our findings suggest that integrins via ILK prevent unfavorable oxidative damage through the regulation of mitochondrial trafficking. This may also provide an additional cell-biological explanation as to how blocking the functions of integrins can sensitize cancer cells to therapeutic modalities, including IR (see also below)^13–16^.

**Fig. 8:**
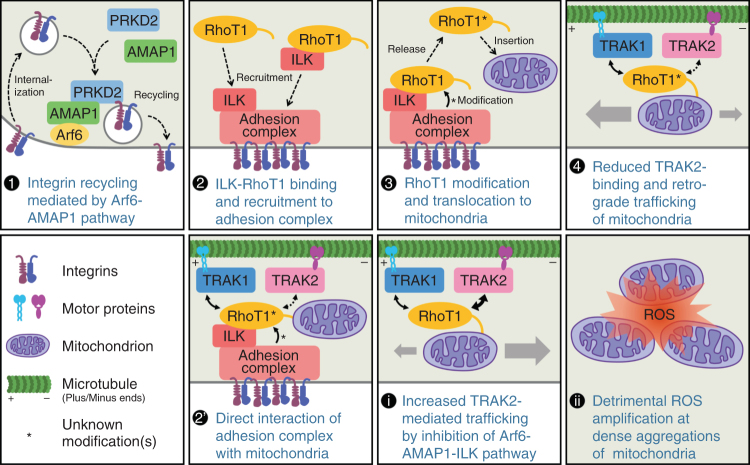
A schematic model of mitochondrial distribution regulated by the Arf6–AMAP1–ILK pathway. Integrin recycling mediated by the Arf6–AMAP1 pathway, which is activated in invasive cancer cells, is essential for the formation of mature FAs (1). The ILK–RhoT1 complex formed at or recruited to FAs (2) might interact with some component(s) of the adhesion complex, which causes alteration of the molecular properties of RhoT1. RhoT1 modified as such would be released and inserted into the mitochondrial outer membrane (3), and negatively regulates the interaction between RhoT1 and TRAK2, leading to relatively increased anterograde trafficking of mitochondria (4). Another possibility is that RhoT1 anchored to the mitochondrial outer membrane may interact with ILK, which is diffusely localized in the cytoplasm, and the above modification(s) may occur when mitochondria reach in close proximity to the mitochondria (2′). Disruption of these mechanisms reduces invasiveness and enhances the molecular interaction between RhoT1 and TRAK2, which preferentially mediates mitochondrial retrograde trafficking (i). The mitochondrial aggregation that is thus caused tends to result in ROS amplification via a RIRR-like mechanism (ii)

The activation of integrin-mediated signaling is closely associated with the formation of FAs or similar multiprotein complexes^16,48^, which has been observed not only in conventional two-dimensional cultures, but also in more physiological three-dimensional cultures^52,59^. Formation of mature FAs may be both a cause and a result of cell movement: nascent adhesions are first formed at the cell periphery, where they undergo a relatively rapid turnover, and then mature as solid structures in response to and to generate forces^51,52^. Our data suggest that ILK localized at FAs reduces mitochondrial retrograde trafficking mediated by the association of RhoT1 and TRAK2, whereas the RhoT1–TRAK1 interaction, which is essential for anterograde trafficking in MDA-MB-231 cells, was not affected. The frequent detection of the ILK–RhoT1 interaction in the cytoplasm and at FAs, irrespective of the colocalization of mitochondria (Supplementary Fig. 10), strongly suggests that “cytoplasmic RhoT1” is the major binding partner of ILK. A recent study showed that some splice variants of RhoT1 can also localize at peroxisomes through the transport process mediated by the interaction with Pex19p^60^, which takes place at the cytoplasm^61^. These facts suggest that RhoT1 might interact with other proteins as well, including ILK, in the cytoplasm and FAs. ILK may bridge RhoT1 to some component(s) of the adhesion complex, which may mediate some modifications on RhoT1 that in turn inhibit its association with TRAK2. Mature FAs containing ILK may thus “signal” to mitochondria to enable directional relocation toward the leading edge of moving cells (Fig. 8).

Our data also suggest that the link between integrins via FAs and mitochondrial trafficking is pivotal for cancer invasion (Fig. 6). It has been demonstrated that the localized production of ATP by mitochondria at the leading edges is crucial for cell movements^3,4^. On the other hand, the regulation of cellular adhesion and motility is also modulated by ROS in cancers^7^. Several key players of cell movements, such as Src and FAK, are known to be directly or indirectly regulated by ROS^7,62,63^, implying that localized ROS production by mitochondria also has important roles in cell movements. However, such utilization of mitochondrial ROS may also increase the risk of RIRR-like ROS amplification unless mitochondrial positioning is properly regulated.

In addition to ATP and ROS, the local production of various other metabolic intermediates by mitochondria may also regulate FAs and/or invasive activity. Recently, we have shown that regulation of the Arf6-based pathway is mediated by the mevalonate pathway (MVP)^24^. Given that MVP is able to utilize mitochondria-derived citrate as a precursor, the presence of mitochondria at the cell periphery might be favorable for local activation of the Arf6-based pathway. As such, there might be a positive feedback loop involving mitochondrial metabolism and integrin functions in the context of cell movement. On the other hand, we previously reported that mitochondrial ATP synthesis is required for the maintenance of polarized acinar structures formed by non-malignant mammary epithelial cells, whereas ATP production in the glycolytic pathway is required for loss of polarity in breast cancer cells^64^. Collectively, these findings further highlight the importance of the context-dependent regulation and/or compartmentalization of intracellular metabolism as potential determinants of cellular functions and phenotypes. In this regard, the association between mitochondrial trafficking/positioning and morphological and phenotypical changes in normal cells, such as epithelial–mesenchymal transition (EMT), might be of particular interest.

In accordance with the above notion, the regulatory mechanisms of mitochondrial distribution linked with β1-integrins appear to be preferable for highly invasive cancer cells (see Fig. 7 and Supplementary Fig. 3). Unexpectedly, the knockdown of TRAK1 did not notably affect the velocity of two-dimensional random migration, whereas it clearly hampered cell invasion. These results suggest that TRAK1-mediated mitochondrial trafficking is favorable for more directional and persistent movements, such as cancer invasion, in which cells have to move through the fine meshwork of matrices to reach their destination. The expression of TRAK proteins appears to be augmented via post-transcriptional regulation in highly invasive cancers, similarly to AMAP1 and Arf6^26,27^, whereas weakly invasive cancers may possibly use other adaptors for mitochondrial trafficking as well. The preferential utilization of a TRAK-based machinery might be associated with EMT, which is intimately linked with the invasive activity of cancer cells^10,11^. More precise mechanisms as to how cancer cells reconnect mitochondrial dynamics to the invasion machinery, including how RhoT-TRAK interaction is regulated by ILK at FAs, also warrant additional investigation, although these issues are far beyond the scope of our present study.

Recurrence associated with the acquisition of invasiveness is a serious unresolved problem in breast cancer radiotherapy^65^. Previous studies have shown that some integrin heterodimers are involved in the survival and/or acquisition of invasiveness in cancer cells after IR treatment^17–21^. The simultaneous reduction of invasiveness and ROS tolerance by targeting Arf6–AMAP1 or associated pathways, such as MVP, which may have some advantages compared to directly targeting integrins themselves^29^, might provide a novel strategy for improving the efficacy of radiotherapy, and also other ROS-mediated therapies^6,9^.

Previous studies collectively indicate that properties of cancer cells, such as EMT and invasion, stem cell-like properties, resistance against therapies including IR, and tolerance to ROS are intimately interconnected^10,11,66,67^. Our findings may provide a new mechanistic explanation as to how EMT and the invasion of cancer cells are associated with their resistance to ROS-mediated cancer therapy. Cancer stem cells often show lower intracellular levels of ROS^66,67^, and asymmetric apportioning of mitochondria in the process of mitosis, based on their age, is also suggested to be important for the maintenance of stem cell-like properties^68^. Future studies are required to clarify whether the Arf6–AMAP1–ILK axis also links to the stem cell-like properties of cancer cells, which may further extend our understanding of the intimate interconnection between the malignant properties of cancers.

## Methods

### Cell culture

Human breast cancer cells were obtained from American Type Culture Collection and expanded following their instructions. For the experiments in this study, all breast cancer cells, except MDA-MB-436 and MCF7, were cultured in a 1:1 mix of Dulbecco’s Modified Eagle’s medium (DMEM) and RPMI1640 (Sigma) supplemented with 10% fetal calf serum (FCS, HyClone), 5% Nu-Serum (Corning), and 1 mM l-glutamine. MDA-MB-436 and MCF7 cells were cultured in DMEM supplemented with 10% FCS. For functional analyses, cells were cultured on plastic or glass bottom dishes (Advanced TC series, Greiner Bio One) coated with 10 μg/mL collagen I. 293T cells were cultured in DMEM supplemented with 10% FCS. All cell lines were regularly tested for mycoplasma contamination with the MycoAlert™ Mycoplasma Detection Kit (Lonza) and/or staining with Hoechst 33342.

### Reagents and antibodies

Chemical reagents were as follows: TMRM (Thermo Fisher Scientific), MitoPY1 (Sigma-Aldrich), Hoechst 33342 (Setareh Biotech), DRAQ5 (BioStatus), A/C Heterodimerizer (equivalent to Rapalog AP21967, Takara Bio), EUK-134 (Cayman Chemical), and Z-VAD-FMK (R&D Systems). Monoclonal or polyclonal antibodies used in this study are described in Supplementary Tables 1 and 2.

### siRNA and transfection

The siRNA duplexes were chemically synthesized by Japan Bio Services or Hokkaido System Science. Sequences were as follows: si-NC (negative control), 5′-GCGCGCUUUGUAGGAUUCGdTdT-3′ and 5′-CGAAUCCUACAAAGCGCGCdTdT-3′; si-AMAP1 #1, 5′-GACCUGACAAAAGCCAUUAdTdT-3′ and 5′-UAAUGGCUUUUGUCAGGUCdTdT-3′; si-AMAP1 #2, 5′-GCUACCCAGUGUGAAGAUCdTdT-3′ and 5′-GAUCUUCACACUGGGUAGCdTdT-3′; si-Arf6 #1, 5′-GCACCGCAUUAUCAAUGACCGdTdT-3′ and 5′-CGGUCAUUGAUAAUGCGGUGCdTdT-3′; si-Arf6 #2, 5′-ACGUGGAGACGGUGACUUAdTdT-3′ and 5′-UAAGUCACCGUCUCCACGUdTdG-3′; si-PRKD2 #1, 5′-CUGCAAGUUUAACUGUCACAAdTdT-3′ and 5′-UUGUGACAGUUAAACUUGCAGdTdT-3′; si-PRKD2 #2, 5′-GGAAAUUCCGCUGUCAGAAdTdT-3′ and 5′-UUCUGACAGCGGAAUUUCCdTdT-3′; si-ILK #1, 5′-UGACAUUGUCGUGAAGGUGdTdT-3′ and 5′-CACCUUCACGACAAUGUCAdTdT-3′; si-ILK #2, 5′-GGUGCUGAAGGUUCGAGACdTdT-3′ and 5′-GUCUCGAACCUUCAGCACCdTdT-3′; si-TRAK1 #1, 5′-AGUUAAGAGCCGACACCAUdTdT-3′ and 5′-AUGGUGUCGGCUCUUAACUdTdA-3′; si-TRAK1 #2, 5′-GACGAAGUGUACUGCCUUAdTdT-3′ and 5′-UAAGGCAGUACACUUCGUCdCdA-3′. The siRNAs targeting human TRAK2 were purchased from Thermo Fisher Scientific. Cells were transfected with siRNA duplexes using Lipofectamine RNAiMAX (Thermo Fisher Scientific), according to the reverse transfection method provided by the manufacturer. Assays were started 48 h after the transfection, unless otherwise described.

### Plasmid DNA construction and transfection

The backbone of pPB, a piggyBac transposon-based vector, was constructed as follows. pBluescript II SK(+) vector (Stratagene) was digested with BssHII sites, and chicken hypersensitivity site 4 (cHS4) insulators flanking the 5′- and 3′-inverted terminal repeat sequences (ITRs) of piggyBac transposon and some restriction enzyme cleavage sites, including *Asc*I, *Not*I, and *Pac*I, were inserted. For the stable expression vectors, an RNA polymerase II promoter, such as CMV7, UbqC, PGK, or a hybrid of the EF1α and HTLV promoters with the upstream CMV enhancer (CEH), multiple cloning sites (MCS), and the HSV TK polyA sequence were tandemly inserted into the cleavage sites. An internal ribosome entry site (IRES) of EMCV and either of the selection marker genes encoding puromycin N-acetyltransferase (pac) or blasticidin S deaminase (bsr) were inserted between the MCS and polyA sequences, resulting in pPB-CMV7/UbqC/CEH-MCS-IP/IB vectors. For the construction of a doxycycline-inducible vector, the TRE3G promoter (Clontech), the MCS, a synthetic polyA sequence, a G-rich sequence from the extension of the β-actin gene, cHS4, the CEH promoter, reverse tetracycline transactivator (rtTA3) cDNA, IRES, pac cDNA, and the HSV TK polyA sequence were tandemly inserted in the cleavage sites, resulting in the pPB-TRE3G-MCS-CEH-rtTA3-IP vector. For construction of GST-fusion proteins, the CMV promoter of the pcDNA3.1 His C vector (Invitrogen) was replaced with the CEH promoter connected to GST cDNA (see below), resulting in the pCEHG vector.

cDNAs encoding mEmerald, tdTomato, mTagBFP2, roGFP2, KillerRed, GST, rtTA3, and ActA MTS were obtained by gene synthesis services. DNA sequences that encode signal peptides targeting intracellular compartments, such as the cytoplasm (cyto), nucleus (nuc), mitochondria (mito), or peroxisomes (perox) were fused on demand. Amino acid sequences of the targeting signal peptides were as follows: cyto, LPPLERLTLD; nuc, LRSRADPKKKRKVDPKKKRKVDPKKKRKVGSTGSR; mito, SVLTPLLLRGLTGSARRLPVPRAKIHSLGDLSVLTPLLLRGLTGSARRLPVPRAKIHSLGD; and perox, SKL. cDNAs encoding human catalase, SOD2, FKBP, FRB, BICD2N, RhoT1, ILK, TRAK1, and TRAK2 were amplified by PCR from the first strand cDNA of MDA-MB-231 cells, which was obtained by SuperScript IV reverse transcriptase (Thermo Fisher Scientific). The catalase cDNA were fused with a DNA sequence encoding the mitochondria-targeting peptide (MLFNLRILLNNAAFRNGHNFMVRNFRCGQPLQ), resulting in mtCAT cDNA. A DNA sequence encoding the HA tag and the ActA MTS cDNA were fused to the N- and C terminus, respectively, of two tandem FKBP cDNAs. The T2098L mutation was introduced into the FRB cDNA by PCR (FRB*), and a DNA sequence encoding the V5 tag and the BICD2N cDNA were fused to the N terminus of the FRB* cDNA. DNA sequences encoding V5 or Xpress tags were fused to the N terminus of RhoT1 or TRAK1/2 cDNAs, respectively. cDNAs encoding ILK (full length and/or fragments) were subcloned into the pCEHG vector or fused to the C terminus of mEmerald.

Arf6, AMAP1, or PRKD2 cDNAs refractory to siRNAs (#1 sequence of each) have been constructed previously^26,28^. Target sequences were replaced as follows: 5′-AAGACCTGACAAAAGCCATTA-3′ to 5′-AGGATTTAACTAAGGCGATAA-3′ (si-AMAP1 #1), 5′-GCACCGCATTATCAATGACCG-3′ to 5′-TCATAGGATAATTAACGATAG-3′ (si-Arf6 #1), 5′-TTGTGACAGTTAAACTTGCAG-3′ to 5′-TTATGGCAATTGAATTTACAA-3′ (si-PRKD2 #1). To obtain refractory ILK cDNA, 5′-TGACATTGTCGTGAAGGTG-3′ (the target sequence of #1 siRNA) was replaced to 5′-CGATATCGTGGTCAAAGTC-3′ by a PCR-based method. An HA tag was fused to the C terminus of Arf6 and AMAP1 refractory cDNAs. Xpress and V5 tags were fused to the N terminus of PRKD2 and ILK refractory cDNAs, respectively.

For transient or stable gene expression, cDNAs were subcloned into the pcDNA3 vector (Invitrogen) or pPB vectors, respectively. Transient transfection was performed by Viafect (Promega) following the manufacturer’s instructions. For sequential transfection, siRNA was first transfected as described above, and then with plasmid DNA after 24 h. Assays were started 24 h after plasmid DNA transfection. For stable transfection, pPB vectors were cotransfected with a plasmid encoding hyperactive piggyBac transposase^69^. Cells were selected by puromycin (1 μg/mL) and/or blasticidin S (5 μg/mL) for 7 days. Assays were performed after culturing the cells in the absence of antibiotics for several days. For the production of GST-fusion proteins, ILK cDNAs encoding full length or fragments, which were subcloned into the pCEHG vector, were transiently transfected.

### Protein extraction, western blotting, and quantification

For protein extraction, cells on culture plates were lysed in NP-40 lysis buffer (1% NP-40, 150 mM NaCl, 20 mM Tris-HCl [pH 7.4], 5 mM EDTA, protease inhibitor cocktail set I, and phosphatase inhibitor cocktail set I [Calbiochem]), after washing with ice-cold phosphate-buffered saline (PBS). For TRAK1 blotting, cells were collected by trypsinization, washed with PBS and then lysed in lysis buffer, to reduce non-specific background bands. For western blotting, 4 × NuPAGE LDS Sample Buffer (Thermo Fisher Scientific) and 2-mercaptoethanol (2-ME) was added to the cell lysates, and the samples were incubated at 70 °C for 10 min. The samples were loaded onto NuPAGE Bis-Tris Gel (Thermo Fisher Scientific), and proteins on the gel were transferred onto Immobilon-FL polyvinylidene difluoride membranes (Millipore). The membranes were blocked with Odyssey Blocking Buffer (LI-COR) or 5% skim milk in PBS, and then incubated with primary antibody solution at 4 °C overnight. After washing with Tris-buffered saline containing Tween 20 (TBST), the membrane was incubated with IRDye secondary antibodies (LI-COR) at room temperature (RT) for 1 h, washed again with TBST and PBS, then scanned with Odyssey Imaging System (LI-COR).

To analyze relative expression levels of the TRAK1/2 protein, densitometry of the western blots was performed using ImageJ software (NIH, version 1.51n). The intensity of TRAK1 and TRAK2 blots were normalized with that of β-actin in each cell line, and the values were normalized using the average value from all of the cell lines used in the same experiment. The results are shown as the mean ± SEM for three independent experiments. All uncropped scans of western blot are shown in Supplementary Fig. 11.

### Immunoprecipitation and GST-pulldown assay

For immunoprecipitation (IP) of endogenous ILK, cell lysates (500 μg each) were incubated with an anti-ILK rabbit mAb or nonimmune rabbit IgG, together with protein A sepharose beads (5 μL bed volume, GE Healthcare) at 4 °C for 4 h with gentle agitation. After washing the sepharose beads 4 times with NP-40 buffer, 1 × NuPAGE LDS Sample Buffer containing 2-ME was added, and the mixture was incubated at 70 °C for 10 min. Western blotting of ILK and RhoT1 was performed with mAbs to avoid detection of the rabbit mAb used for IP. For co-IP of V5-RhoT1 and Xpress-TRAK1/2, cell lysates (300 μg each) were incubated with an anti-V5 epitope antibody conjugated with agarose beads (5 μL bed volume, Wako) at 4 °C for 2 h with gentle agitation. Agarose beads were washed four times with NP-40 buffer, and transferred onto a Micro-Spin Column (Thermo Fisher Scientific). After removing the NP-40 buffer by brief centrifugation, 1 × NuPAGE LDS Sample Buffer (not containing 2-ME) was added, and incubated at 70 °C for 10 min. The eluate was collected by centrifugation, and further incubated at 70 °C for 10 min after addition of 2-ME. For preparation of GST-fusion proteins, 293T cells were transfected with pCEHG vectors encoding ILK cDNAs (full length or fragments), using Polyethylenimine “MAX” (Polysciences). Cells were lysed in NP-40 buffer, and the lysates were incubated with Glutathione Sepharose 4B beads (GE Healthcare) at 4 °C for 2 h, with gentle agitation, and then extensively washed with lysis buffer. For the pulldown assay, GST-fusion proteins (5 μg each) were incubated with lysate (500 μg) of 293 T cells expressing V5-RhoT1, at 4 °C for 2 h with gentle agitation. Beads were washed 4 times with NP-40 buffer, and incubated at 70 °C for 10 min after addition of 4 × LDS Sample Buffer and 2-ME. Samples were separated by NuPAGE and analyzed by western blot, as above.

### Examination of intracellular ROS levels

To quantify intracellular ROS levels, cells plated on collagen I-coated glass bottom dishes, and supplemented with phenol red-free medium, were incubated with 2.5 μg/mL Hoechst 33342 and 10 μM dihydroethidium (DHE) for 30 min. EUK-134 (100 μM) or Rapalog (500 μM) was added to the cells 24 h before the assay. Three fluorescent images, containing at least 35 cells each, were randomly taken using a Leica TCS SP8 confocal laser-scanning microscope (Leica Microsystems), equipped with a heater and CO_2_ incubator for live cell imaging. For visualization of Hoechst staining, a 405 nm laser and 410–574 nm spectral detection were used. For DHE, a 552 nm laser and a 580–755 nm spectral detection were used. As excitation at 552 nm is not selective for superoxide-specific oxidation products of DHE^70^, the fluorescence reflects general/non-specific oxidation. DHE fluorescence intensity per cell was calculated in each image, and average values were normalized by that of non-treated parental cells. The results are shown as mean ± SEM for three independent experiments.

For the brief examination of redox states in subcellular compartments, cells stably expressing roGFP2 sensors were cultured on collagen I-coated glass bottom dishes with phenol red-free medium. Fluorescent images were randomly taken by a Nikon A1R confocal microscope (Nikon Instech) equipped with a heater and CO_2_ incubator, using 405 and 488 nm lasers for excitation, and a 500–550 nm spectral detection, respectively. The 405/488 nm excitation ratio was calculated and shown as a heatmap using NIS-Elements AR (version 4.11, Nikon Instech) software that accompanied the microscope.

### Immunofluorescence staining and microscopy

For quantification of intracellular mitochondrial distribution, cells plated on collagen I-coated glass bottom dishes were first fixed with 2% paraformaldehyde (PFA) at 37 °C for 10 min, by directly adding a 1/17.5 volume of 37% PFA to the culture medium. Rapalog (500 μM) or β1-integrin inhibitory antibody AIIB2 (2.5 μg/mL) was added to the cells 24 h before PFA fixation. After washing once with PBS, cells were further fixed with methanol at −20 °C for 5 min. Cells were incubated with MAXblock Blocking Medium (Active Motif) for 2 h at RT, washed trice with PBS, and then incubated with the anti-α-tubulin Ab (1:400) for 1 h at RT. Cells were further incubated with Alexa Fluor 568-conjugated anti-mouse IgG Ab (1:400) and then Alexa Fluor 488-conjugated anti-TOMM20 mouse Ab (1:1000) and Alexa Fluor 647-conjugated anti-γ-tubulin Ab (1:400), for 1 h each at RT. Antibodies were diluted in PBS containing 5% bovine serum albumin (BSA), and cells were washed trice with PBS after each antibody incubation. Finally, cells were incubated with Hoechst 33342 (2.5 μg/mL in PBS) for 10 min at RT and washed trice with PBS. Immunostaining of catalase, SOD2, HA-tagged FKBP-MTS, and V5-tagged BICD2N-FRB* was also performed essentially in the same way, using specific antibodies diluted at 1:200, 1:200, 1:800, and 1:400, respectively. For immunostaining of ILK, the procedure was essentially the same, except that the PFA fixation step was omitted. Antibodies used were anti-α-tubulin (1:400) and anti-ILK (1:200) antibodies, for the first incubation; Alexa Fluor 488-conjugated anti-rabbit IgG (1:400) and Alexa Fluor 568-conjugated anti-mouse IgG (1:400) antibodies, for the second incubation. For immunostaining of γ-H2AX, fixation and permeabilization were performed by incubation with 4% PFA in PBS and then Triton X-100 in PBS, at RT for 10 min each. Immunostaining of γ-H2AX was performed with a specific antibody (1:200), and cells were stained with Hoechst 33342, as above.

Immunofluorescence images were taken by a Leica TCS SP8 confocal microscope, using optimal settings for the lasers (405, 488, 552, and 638 nm) and spectral detection. When applicable, Adaptive Focus Control was activated to maintain the focus position.

### Proximity ligation assay

To visualize the subcellular localization of the ILK–RhoT1 interaction, MDA-MB-231 cells were stably transfected with pPB vectors encoding mEmerald-ILK and V5-RhoT1. To avoid mislocalization of ILK by its excessive expression, mEmerald-ILK cDNA was placed downstream of the TRE3G promoter, and expression was induced by the addition of 10 nM doxycycline 1 h after plating. After 48 h, cells were sequentially fixed with 2% PFA and −20 °C methanol, and blocked with MAXblock as described above. After washing in PBS, cells were incubated with an anti-ILK mouse mAb (1:50) and anti-V5 rabbit mAb (1:50) in 5% BSA/PBS for 16 h at 4 °C. PLA reactions were performed using Duolink® In Situ PLA Probe Anti-Mouse PLUS, Anti-Rabbit MINUS, and Detection Reagents Orange (Sigma), following the manufacturer’s instructions. After the ligation and amplification reactions, cells were further incubated with the anti-TOMM20 antibody conjugated with Alexa 405 (1:100) and DRAQ5 (1:5000) in Duolink Antibody Diluent (supplied in the kit), to visualize mitochondria and nuclei, respectively. Specificity of the antibodies used for PLA was confirmed by immunostaining performed at the same concentrations.

### Quantification of mitochondrial distribution

For semi-automated measurement of the mitochondrial distribution using MetaMorph software (Molecular Devices), an original Journal (macro) was developed. The procedure of the macro was as follows: after loading the multi-channel fluorescence images containing Hoechst/TOMM20/α-tubulin/γ-tubulin staining, thresholds for TOMM20 and α-tubulin fluorescence were automatically or manually determined. A single cell to be analyzed was manually selected, and the peripheral edge of the α-tubulin staining was automatically detected, and defined as “boundary #5”. The center of the cell was manually determined based on γ-tubulin staining, which indicates the location of the MTOC. Boundary #5 was shrunk by 20%, 40%, 60%, and 80% to generate boundaries #4, #3, #2, and #1, respectively, and all the boundaries were aligned with the center. The area in boundary #1 was named as region #1, and those between boundaries #1 and #2, #2 and #3, #3 and #4, and #4 and #5 as regions #2, #3, #4, and #5, respectively. The fluorescence intensity of TOMM20 included in each region was measured, and the ratio to the total intensity was calculated (see also Fig. 1h). For each independent experiment, at least 34 cells were measured, so that the total number in 3 independent experiments would be more than 100. Cells without clear MTOC identification were omitted from the quantification. The average values for each region were calculated, and the results were shown as the mean ± SEM for three independent experiments. The code for the macro is available from the corresponding author upon request.

### Measurement of cell death

Cell death in a steady state or after IR treatment was measured by CytoTox-Glo (Promega), a luciferase-based system which measures the dead/live ratio of cells, following the manufacturer’s instructions. Dead/live ratios were measured 96 h after reverse transfection of siRNAs, in which 7000 cells were plated on collagen I-coated 96 well plates. Cells were treated with 100 μM EUK-134 or vehicle 48 h after transfection/plating. For the assay with IR treatment, cells were exposed to 2 or 8 Gy X-ray 48 h after transfection/plating, and cell death was analyzed 48 h after the IR treatment. For the caspase inhibition, cells were treated with 50 μM Z-VAD-FMK 36 h after transfection/plating. For the assay using cells stably expressing FKBP-MTS and BICD2N-FRB*, cells were treated with 500 μM Rapalog or vehicle 24 h after transfection/plating. For each experiment, average dead/live ratios were determined from triplicated samples. The results are shown as the mean ± SEM for three independent experiments.

### Examination of relative OCR

Cells were collected by trypsinizaion 48 h after siRNA transfection, and 40,000 cells suspended in phenol red-free medium were plated on collagen I-coated 96 well plates. After 24 h, MitoXpress-Xtra (Luxel BioSciences), a fluorescent probe for oxygen measurement, was added, and the culture medium was covered with mineral oil to block exposure to air. Air-saturated water and deoxygenated water (containing 100 mg/mL sodium sulfite) were included as positive (100%) and negative (0%) controls, respectively. Culture medium, not containing the fluorescent probe, was also included for background correction. Following the manufacturer’s instructions, time-resolved fluorescence was measured every 10 min for 18 h, using an Infinite 200 microplate reader (TECAN). For each time point, average oxygen concentration of duplicated samples were determined. OCR was estimated by collinear approximation, and normalized with that of parental cells. The results are shown as the mean ± SEM for three independent experiments.

### Quantitative PCR

Multi-channel quantitative PCR was performed using LightCycler Nano and FastStart Essential DNA Probes Master (Roche), with specific primer pairs and hydrolysis probes for the targets. As a function of mitochondrial biogenesis, ratios of mitochondrial to nuclear DNA were measured. Total DNA of cells treated with siRNAs were obtained using NucleoSpin Tissue (Macherey-Nagel). Primers and probes were as follows: For nuclear DNA, primer-1, 5′-TCTTCTGGACTGTGAACCTGTG-3′; primer-2, 5′-CTGGTGGGAAAGATGACCAC-3′; and probe, Universal Probe Library (UPL) #59 (Roche). For mitochondrial DNA, primer-1, 5′-CTTCTGGCCACAGCACTTAAAC-3′; primer-2, 5′-GCTGGTGTTAGGGTTCTTTGTTTT-3′; and probe, 5′-/HEX/ATCTCTGCC/ZEN/AAACCCC/IABkFQ/-3′ (synthesized by IDT). The default settings were used for amplification and detection. The average ratio of mitochondrial to nuclear DNA was calculated from the results of triplicated reactions in each experiment, and the values were normalized to that of non-treated cells. The results were shown as box-and-whisker plots for three independent experiments, because some data did not pass the normality test.

For measurement of TRAK1/2 mRNA expression, total RNA of breast cancer cells was extracted using NucleoSpin RNA (Macherey-Nagel). To obtain first strand cDNA pools, reverse transcription was carried out using SuperScript IV (Thermo Fisher Scientific) with a random hexamer and oligo dT primer, following the manufacturer’s instructions. Primers and probes were as follows: For TRAK1, primer-1, 5′-CCAGTTCAGCACCCAGAGAC-3′; primer-2, 5′-GATGCGACAGGTGGTGAAG-3′; and probe, UPL #39 (Roche). For TRAK2, primer-1, 5′-GATCTTCCTGCCACCCATTA-3′; primer-2, 5′-TGTGCACGACAGGCACTT-3′; probe, 5′-GATGCGACAGGTGGTGAAG-3′; UPL #62 (Roche). For ACTB (β-actin), UPL Human ACTB Gene Assay (Roche), which includes primers and a probe, was used. Default settings were used for amplification and detection, except that the annealing condition was changed to 58 °C for 60 s, and the reaction was continued up to 60 cycles. As an internal control, a mixture of all the samples was also analyzed. The average ratio of TRAK1/2 to β-actin mRNA levels was calculated from the results of triplicated reactions in each experiment, and the values were normalized to that of the internal control. The results are shown as the mean ± SEM for three independent experiments.

### Cell-cycle analysis

MDA-MB-231 cells were stably transfected with pPB vectors encoding Fucci probes^44^. Cells grown on a collagen I-coated glass bottom dish supplemented with phenol red-free medium, were exposed to 2 or 8 Gy X-ray 48 h after siRNA transfection. At the indicated time points, cells were incubated with 2.5 μg/mL Hoechst 33342, and three fluorescence images were randomly taken using a Leica TCS SP8 confocal laser-scanning microscope equipped with a heater and CO_2_ incubator. Percentages of the cells expressing a G1 probe alone was calculated, and the results were shown as the mean ± SEM for three independent experiments.

### Quantification of ROS production in mitochondria

MDA-MBA-231 cells, either parental or expressing KillerRed-NES and mTagBFP2-mito, were cultured on collagen I-coated glass bottom dishes, using phenol red-free medium (Thermo Fisher Scientific). To avoid unexpected effects by stable expression, KillerRed-NES cDNA was placed downstream of the TRE3G promoter, and its expression was induced by the addition of 10 nM doxycycline 1 h after plating. After 24 h, parental cells were incubated with 10 nM TMRM and 5 μM MitoPY1 for 30 min, and cells expressing KillerRed-NES and mTagBFP2-mito were incubated with 5 μM MitoPY1 alone for 30 min. Using the FRAP system of a Leica TCS SP8 confocal microscope equipped with a heater and CO_2_ incubator, a single cell captured in the view was exposed to a 552 nm laser with the predetermined power density, to induce ROS production by TMRM or KillerRed. Fluorescence of MitoPY1 (ROS), and TMRM or mTagBFP2 (mitochondria) was visualized by scanning with 488, 552, and 405 nm lasers at minimal power density, respectively, before and after the ROS induction. To avoid fluorescence saturation, TMRM excitation intensity (for visualization of mitochondria) was deliberately reduced in cells with a dense mitochondrial network. Change of the MitoPY1 and mTagBFP2 fluorescence was observed every 20 s after the ROS induction, up to 200 s.

For quantification, obtained FRAP data were analyzed with the associated software (LAS X). For the assay using parental cells and TMRM, background and measurement ROIs were determined so that the former did not include cell bodies or debris, and the latter included entire cell body of the cell of interest throughout the time-course experiment. MitoPY1 fluorescence in each time point (*F*_t_) was quantified by subtracting the total fluorescence of the background ROI from that of the measurement ROI. The initial MitoPY1 fluorescence before ROS induction (*F*_i_), which reflects basal ROS production, was further subtracted from the fluorescence values right after ROS induction (*F*_0_) and each time point thereafter (*F*_20–200_). The *F*_t_ values thus corrected were normalized to the corrected *F*_0_ value. For the assay using cells expressing KillerRed-NES and mTagBFP2-mito, background and measurement ROIs were determined so that the former did not include cell bodies or debris, and the latter included sparse or dense mitochondrial networks, throughout the time-course experiment. MitoPY1 fluorescence (*F*_t_) as well as mTagBFP2 fluorescence (*M*_t_), the latter of which reflects the number of mitochondria included in the ROI, were quantified at each time point. The initial mTagBFP2 fluorescence (*M*_i_) was assumed to be the same as that right after ROS induction (*M*_0_), since mTagBFP2 appeared to be partially bleached by the 552 nm laser. *F*_t_/*M*_t_, which is MitoPY1 fluorescence per mitochondria, was calculated in each ROI, and *F*_i_/*M*_i_ (i.e., equal to *F*_i_/*M*_0_), was subtracted. Thus, corrected *F*_t_/*M*_t_ values were normalized to the corrected *F*_0_/*M*_0_ value. In each experiment, four each of sparse or dense ROIs were measured, and their average was calculated. For each assay, the results are shown as the mean ± SEM for 10 independent experiments.

### Invasion assay

In vitro invasion activity was analyzed using Biocoat Matrigel chambers (Corning). Cells were collected by trypsinization 48 h after transfection, and 100,000 cells were suspended in the assay medium (a 1:1 mixture of DMEM and RPMI1640 supplemented with 0.1% BSA and 10 ng/mL EGF, but not FCS or Nu-Serum) were loaded on the upper wells of 24-well matrigel chambers, the underside of which was pre-coated with 10 μg/mL collagen I. The lower wells were also filled with the assay medium. For the assay using cells stably expressing FKBP-MTS and BICD2N-FRB*, 500 μM Rapalog was also added to the assay medium, as indicated. To analyze the effects of the various treatments on adhesion and viability, 50,000 cells were plated in 24-well plates pre-coated with 10 μg/mL collagen I. After incubation for 16 h, cells were fixed with 4% PFA in PBS for 10 min at RT. The number of cells that migrated out onto the underside of the membrane (invasion), or attached onto the collagen I-coated plastic surface (adhesion/viability), was scored by staining the cells with 1% crystal violet or Hoechst 33342, respectively. Samples were duplicated in each experiment, and invasions normalized to adhesion/viability were calculated. The invasiveness scores obtained were normalized to those of the control condition (i.e., si-NC-treated cells or cells transfected with empty vectors), and the results were shown as mean ± SEM for three independent experiments.

### Random migration assay

Cells stably expressing tdTomato were seeded on glass bottom dishes coated with 10 μg/mL collagen I. After 48 h, images of randomly chosen fields of view containing at least 10 cells each, were acquired by time-lapse confocal microscopy using Nikon A1R. Each image was acquired every 10 min for 8 h using the multipoint acquisition tool associated with NIS-Elements AR. Perfect Focus System was activated to maintain the focus position. Object tracking was performed using MetaMorph software, and the mean velocity of randomly chosen 10 cells were calculated. The results were shown as mean ± SEM for three independent experiments.

### Statistics and reproducibility

All experiments were repeated at least three times. Normality of the data sets was examined by the Shapiro–Wilk test. For data with a normal distribution, the results are shown as mean values with error bars indicating the SEM. Statistical significance was examined by the two-tailed *t*-test, after the equality of variance was judged by the *F*-test. The *P*-values were adjusted by the Holm–Sidak method, when multiple samples were compared. For data with a non-normal distribution, results were shown as box-and-whisker plots. Statistical significance was examined by the Mann–Whitney *U* test after confirming equal variance by the *F*-test, and *P*-values were adjusted by the Sidak method when multiple samples were compared. No statistical method was used to predetermine sample size. None of the samples were excluded from the experiment, unless there were clear technical mistakes. The experiments were not randomized, but were performed in essentially the same manner. Investigators were not blinded during the experiments and outcome assessment, but had no pre-conceptions. The images shown are representative of more than 10 samples.

### Data availability

Source data for figures are available from the corresponding author upon request.

## Electronic supplementary material

Supplementary Information

## References

[1] Campello S (2006). Orchestration of lymphocyte chemotaxis by mitochondrial dynamics. J. Exp. Med..

[2] Caino MC (2015). PI3K therapy reprograms mitochondrial trafficking to fuel tumor cell invasion. Proc. Natl Acad. Sci. USA.

[3] Schuler MH (2017). Miro1-mediated mitochondrial positioning shapes intracellular energy gradients required for cell migration. Mol. Biol. Cell.

[4] Cunniff B, McKenzie AJ, Heintz NH, Howe AK (2016). AMPK activity regulates trafficking of mitochondria to the leading edge during cell migration and matrix invasion. Mol. Biol. Cell.

[5] Desai SP, Bhatia SN, Toner M, Irimia D (2013). Mitochondrial localization and the persistent migration of epithelial cancer cells. Biophys. J..

[6] Gorrini C, Harris IS, Mak TW (2013). Modulation of oxidative stress as an anticancer strategy. Nat. Rev. Drug Discov..

[7] Liou GY, Storz P (2010). Reactive oxygen species in cancer. Free Radic. Res..

[8] Sullivan LB, Chandel NS (2014). Mitochondrial reactive oxygen species and cancer. Cancer Metab..

[9] Panieri E, Santoro MM (2016). ROS homeostasis and metabolism: a dangerous liason in cancer cells. Cell Death Dis..

[10] Singh A, Settleman J (2010). EMT, cancer stem cells and drug resistance: an emerging axis of evil in the war on cancer. Oncogene.

[11] Shibue T, Weinberg RA (2017). EMT, CSCs, and drug resistance: the mechanistic link and clinical implications. Nat. Rev. Clin. Oncol..

[12] Alexander S, Friedl P (2012). Cancer invasion and resistance: interconnected processes of disease progression and therapy failure. Trends Mol. Med..

[13] Seguin L, Desgrosellier JS, Weis SM, Cheresh DA (2015). Integrins and cancer: regulators of cancer stemness, metastasis, and drug resistance. Trends Cell Biol..

[14] Blandin AF (2015). β1 integrins as therapeutic targets to disrupt hallmarks of cancer. Front. Pharmacol..

[15] Nam JM, Chung Y, Hsu HC, Park CC (2009). Beta1 integrin targeting to enhance radiation therapy. Int. J. Radiat. Biol..

[16] Eke I, Cordes N (2015). Focal adhesion signaling and therapy resistance in cancer. Semin. Cancer Biol..

[17] Eke I (2012). β_1_Integrin/FAK/cortactin signaling is essential for human head and neck cancer resistance to radiotherapy. J. Clin. Invest..

[18] Nam JM, Onodera Y, Bissell MJ, Park CC (2010). Breast cancer cells in three-dimensional culture display an enhanced radioresponse after coordinate targeting of integrin alpha5beta1 and fibronectin. Cancer Res..

[19] Nalla AK (2010). Suppression of uPAR retards radiation-induced invasion and migration mediated by integrin β1/FAK signaling in medulloblastoma. PLoS ONE.

[20] Yao H (2011). Role of α(5)β(1) integrin up-regulation in radiation-induced invasion by human pancreatic cancer cells. Transl. Oncol..

[21] Nam JM (2013). β1-Integrin via NF-κB signaling is essential for acquisition of invasiveness in a model of radiation treated in situ breast cancer. Breast Cancer Res..

[22] Nam JM (2007). CIN85, a Cbl-interacting protein, is a component of AMAP1-mediated breast cancer invasion machinery. EMBO J..

[23] Morishige M (2008). GEP100 links epidermal growth factor receptor signalling to Arf6 activation to induce breast cancer invasion. Nat. Cell Biol..

[24] Hashimoto A (2016). P53- and mevalonate pathway-driven malignancies require Arf6 for metastasis and drug resistance. J. Cell. Biol..

[25] Hashimoto S (2016). Lysophosphatidic acid activates Arf6 to promote the mesenchymal malignancy of renal cancer. Nat. Commun..

[26] Hashimoto S (2004). Requirement for Arf6 in breast cancer invasive activities. Proc. Natl Acad. Sci. USA.

[27] Onodera Y (2005). Expression of AMAP1, an ArfGAP, provides novel targets to inhibit breast cancer invasive activities. EMBO J..

[28] Onodera Y (2012). Rab5c promotes AMAP1-PRKD2 complex formation to enhance β1 integrin recycling in EGF-induced cancer invasion. J. Cell. Biol..

[29] Onodera Y, Nam JM, Sabe H (2013). Intracellular trafficking of integrins in cancer cells. Pharmacol. Ther..

[30] Zorov DB, Filburn CR, Klotz LO, Zweier JL, Sollott SJ (2000). Reactive oxygen species (ROS)-induced ROS release: a new phenomenon accompanying induction of the mitochondrial permeability transition in cardiac myocytes. J. Exp. Med..

[31] Zorov DB, Juhaszova M, Sollott SJ (2014). Mitochondrial reactive oxygen species (ROS) and ROS-induced ROS release. Physiol. Rev..

[32] Park J, Lee J, Choi C (2011). Mitochondrial network determines intracellular ROS dynamics and sensitivity to oxidative stress through switching inter-mitochondrial messengers. PLoS ONE.

[33] Rong Y, Doctrow SR, Tocco G, Baudry M (1999). EUK-134, a synthetic superoxide dismutase and catalase mimetic, prevents oxidative stress and attenuates kainate-induced neuropathology. Proc. Natl Acad. Sci. USA.

[34] Hanson GT (2004). Investigating mitochondrial redox potential with redox-sensitive green fluorescent protein indicators. J. Biol. Chem..

[35] Dickinson BC, Chang CJ (2008). A targetable fluorescent probe for imaging hydrogen peroxide in the mitochondria of living cells. J. Am. Chem. Soc..

[36] Bulina ME (2006). A genetically encoded photosensitizer. Nat. Biotechnol..

[37] van Spronsen M (2013). TRAK/Milton motor-adaptor proteins steer mitochondrial trafficking to axons and dendrites. Neuron.

[38] Pistor S, Chakraborty T, Niebuhr K, Domann E, Wehland J (1994). The ActA protein of Listeria monocytogenes acts as a nucleator inducing reorganization of the actin cytoskeleton. EMBO J..

[39] Bayle JH (2006). Rapamycin analogs with differential binding specificity permit orthogonal control of protein activity. Chem. Biol..

[40] Lomax ME, Folkes LK, O’Neill P (2013). Biological consequences of radiation-induced DNA damage: relevance to radiotherapy. Clin. Oncol..

[41] Hwang A, Muschel RJ (1998). Radiation and the G2 phase of the cell cycle. Radiat. Res..

[42] Erenpreisa J, Cragg MS (2001). Mitotic death: a mechanism of survival? A review. Cancer Cell. Int..

[43] Bache M (2001). Loss of G2/M arrest correlates with radiosensitization in two human sarcoma cell lines with mutant p53. Int. J. Cancer.

[44] Sakaue-Sawano A (2008). Visualizing spatiotemporal dynamics of multicellular cell-cycle progression. Cell.

[45] Fu MM, Holzbaur ELF (2014). Integrated regulation of motor-driven organelle transport by scaffolding proteins. Trends Cell Biol..

[46] Sheng ZH, Cai Q (2012). Mitochondrial transport in neurons: impact on synaptic homeostasis and neurodegeneration. Nat. Rev. Neurosci..

[47] Varjosalo M (2013). Interlaboratory reproducibility of large-scale human protein-complex analysis by standardized AP-MS. Nat. Methods.

[48] Legate KR, Montañez E, Kudlacek O, Fässler R (2006). ILK, PINCH and parvin: the tIPP of integrin signalling. Nat. Rev. Mol. Cell Biol..

[49] Söderberg O (2006). Direct observation of individual endogenous protein complexes in situ by proximity ligation. Nat. Methods.

[50] Berrier AL, Yamada KM (2007). Cell-matrix adhesion. J. Cell Physiol..

[51] Giannone G, Sheetz MP (2006). Substrate rigidity and force define form through tyrosine phosphatase and kinase pathways. Trends Cell Biol..

[52] Geiger B, Spatz JP, Bershadsky AD (2009). Environmental sensing through focal adhesions. Nat. Rev. Mol. Cell Biol..

[53] van Bergeijk P, Hoogenraad CC, Kapitein LC (2016). Right time, right place: probing the functions of organelle positioning. Trends Cell Biol..

[54] Haga N, Fujita N, Tsuruo T (2003). Mitochondrial aggregation precedes cytochrome c release from mitochondria during apoptosis. Oncogene.

[55] Nazarewicz RR (2007). Tamoxifen induces oxidative stress and mitochondrial apoptosis via stimulating mitochondrial nitric oxide synthase. Cancer Res..

[56] Bonora M, Pinton P (2014). The mitochondrial permeability transition pore and cancer: molecular mechanisms involved in cell death. Front. Oncol..

[57] Song G, Ouyang G, Bao S (2005). The activation of Akt/PKB signaling pathway and cell survival. J. Cell. Mol. Med..

[58] Sulzmaier FJ, Jean C, Schlaepfer DD (2014). FAK in cancer: mechanistic findings and clinical applications. Nat. Rev. Cancer.

[59] Hakkinen KM, Harunaga JS, Doyle AD, Yamada KM (2011). Direct comparisons of the morphology, migration, cell adhesions, and actin cytoskeleton of fibroblasts in four different three-dimensional extracellular matrices. Tissue Eng. Part A.

[60] Okumoto K (2017). New splicing variants of mitochondrial Rho GTPase-1 (Miro1) transport peroxisomes. J. Cell. Biol..

[61] Fujiki Y, Matsuzono Y, Matsuzaki T, Fransen M (2006). Import of peroxisomal membrane proteins: the interplay of Pex3p- and Pex19p-mediated interactions. Biochim. Biophys. Acta.

[62] Ben Mahdi MH, Andrieu V, Pasquier C (2000). Focal adhesion kinase regulation by oxidative stress in different cell types. IUBMB Life.

[63] Giannoni E, Buricchi F, Raugei G, Ramponi G, Chiarugi P (2005). Intracellular reactive oxygen species activate Src tyrosine kinase during cell adhesion and anchorage-dependent cell growth. Mol. Cell Biol..

[64] Onodera Y, Nam JM, Bissell MJ (2014). Increased sugar uptake promotes oncogenesis via EPAC/RAP1 and O-GlcNAc pathways. J. Clin. Invest..

[65] Hayes SB, Freedman GM, Li T, Anderson PR, Ross E (2008). Does axillary boost increase lymphedema compared with supraclavicular radiation alone after breast conservation?. Int. J. Radiat. Oncol. Biol. Phys..

[66] Diehn M (2009). Association of reactive oxygen species levels and radioresistance in cancer stem cells. Nature.

[67] Dong C (2013). Loss of FBP1 by Snail-mediated repression provides metabolic advantages in basal-like breast cancer. Cancer Cell..

[68] Katajisto P (2015). Stem cells. Asymmetric apportioning of aged mitochondria between daughter cells is required for stemness. Science.

[69] Yusa K, Zhou L, Li MA, Bradley A, Craig NL (2011). A hyperactive piggyBac transposase for mammalian applications. Proc. Natl Acad. Sci. USA.

[70] Zhao H, Kalivendi S, Zhang H, Joseph J (2003). Superoxide reacts with hydroethidine but forms a fluorescent product that is distinctly different from ethidium: potential implications in intracellular fluorescence detection of superoxide. Free Radic. Biol. Med..

