# Supplementary material for: Arf6-driven cell invasion is intrinsically linked to TRAK1-mediated mitochondrial anterograde trafficking to avoid oxidative catastrophe
Source: Nat Commun. 2018 Jul 11;9:2682. doi: 10.1038/s41467-018-05087-7 (PMC6041267; doi:10.1038/s41467-018-05087-7)
Supplement: Supplementary file 1 — Supplementary Information [file 41467_2018_5087_MOESM1_ESM.pdf]

SUPPLEMENTARY INFORMATION

Arf6-driven cell invasion is intrinsically linked to TRAK1-mediated mitochondrial anterograde trafficking to avoid oxidative catastrophe

Onodera et al.

Supplementary Table 1. List of antibodies (without conjugation)

Target (Clone)	Company	Catalogue #	Host	Application	Dilution
β-actin (AC-15)	Sigma-Aldrich	A5441	Mouse	WB	1:5000
Arf6 (3A-1)	Santa Cruz Biotechnology	sc-7971	Mouse	WB	1:1000
AMAP1 (2G7)	Sigma-Aldrich	WH0050807M1	Mouse	WB	1:10000
active Caspase-3 (E83-77)	Abcam	ab32042	Rabbit	WB	1:500
Catalase (12C2DB9)	Abcam	ab110292	Mouse	IF	1:200
Catalase	Abgent	AP8623c	Rabbit	WB	1:2000
ILK (3/ILK)	BD Biosciences	611802	Rabbit	WB	1:2000
ILK (4GK)	Cell Signaling Technology	3856	Rabbit	WB	1:10000
				IF	1:200
				IP	1:200
ILK (65.1)	Santa Cruz Biotechnology	sc-20019	Mouse	PLA	1:50
Rab7 (D95F2)	Cell Signaling Technology	9367	Rabbit	IF	1:100
RhoT1 (4H4)	Abnova	H00055288-M01	Mouse	WB	1:1000
SOD2	Proteintech	24127-1-AP	Rabbit	WB	1:2000
				IF	1:200
TRAK1	Abnova	PAB6727	Goat	WB	1:1000
TRAK2	Proteintech	13770-1-AP	Rabbit	WB	1:1000
α-tubulin (DM1A)	Sigma-Aldrich	T6199	Mouse	IF	1:400
β-tubulin	Santa Cruz Biotechnology	sc-9104	Rabbit	WB	1:500
γ-H2AX (JBW301)	Millipore	05-636	Mouse	IF	1:200
GST tag	Bethyl Laboratories	A190-122A	Rabbit	WB	1:5000
HA tag (C29F4)	Cell Signaling Technology	3724	Rabbit	WB	1:2000
				IF	1:1600
V5 tag (1H6)	Medical & Biological Laboratories	M167-3	Mouse	WB	1:1000
V5 tag (D3H8Q)	Cell Signaling Technology	13202	Rabbit	IF	1:400
				PLA	1:50
Xpress tag	Thermo Fisher Scientific	R910-25	Mouse	WB	1:5000

WB: western blot, IF: immunofluorescence, IP: immunoprecipitation, PLA: proximity ligation assay.

Supplementary Table 2. List of antibodies conjugated with fluorescent dyes

Target-Conjugation (Clone)	Company	Catalogue #	Host	Application	Dilution
TOMM20-Alexa Fluor 405 (EPR15581-54)	Abcam	ab210047	Rabbit	IF (for PLA)	1:100
TOMM20-Alexa Fluor 488 (EPR15581-39)	Abcam	ab205486	Rabbit	IF	1:1000
γ-tubulin-Alexa Fluor 647 (TU-30)	Abcam	ab191114	Mouse	IF	1:400
Mouse IgG-Alexa Fluor 488	Abcam	ab150117	Goat	IF (2nd)	1:400
Mouse IgG-Alexa Fluor 568	Abcam	ab175701	Goat	IF (2nd)	1:400
Rabbit IgG-Alexa Fluor 488	Abcam	ab150081	Goat	IF (2nd)	1:400
Rabbit IgG-Alexa Fluor 568	Abcam	ab175695	Goat	IF (2nd)	1:400
Mouse IgG-IRDye 680LT	LI-COR	926-68020	Goat	WB (2nd)	1:20000
Mouse IgG-IRDye 800CW	LI-COR	926-32210	Goat	WB (2nd)	1:10000
Rabbit IgG-IRDye 680LT	LI-COR	926-68021	Goat	WB (2nd)	1:20000
Rabbit IgG-IRDye 800CW	LI-COR	926-32211	Goat	WB (2nd)	1:10000
Goat IgG-Alexa Fluor 790	Abcam	ab175784	Donkey	WB (2nd)	1:20000

WB: western blot, IF: immunofluorescence.

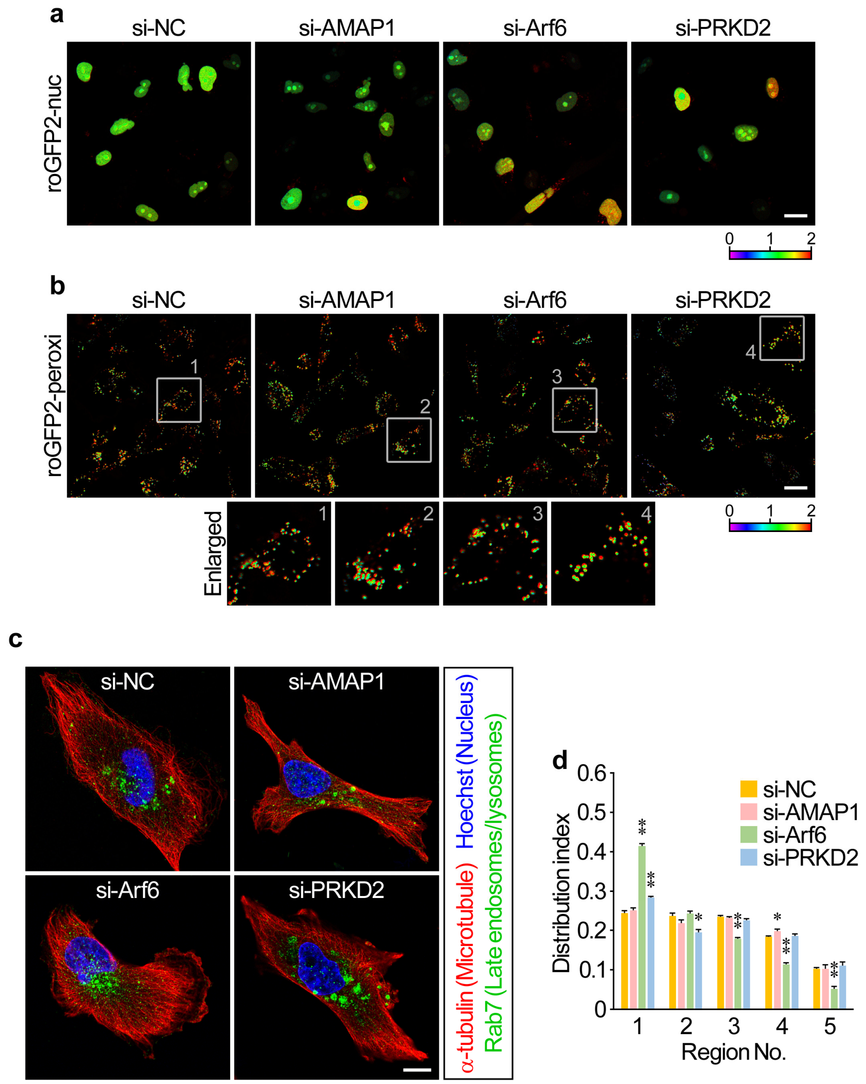

Supplementary Figure 1. Effects of inhibition of the Arf6-AMAP1-PRKD2 pathway on the properties of other organelles.

MDA-MB-231 cells, either parental or stably expressing roGFP2 probes, were transfected with siRNA targeting AMAP1, Arf6, or PRKD2. Subcellular redox states analyzed by roGFP2 localized at nuclei (nuc, **a**) or peroxisomes (peroxi, **b**). The fluorescence ratios of 405 nm to 488 nm excitation is indicated by the heat map. The numbered white squares in (**b**) indicate the enlarged areas shown beneath. Bar, 20 µm. (**c**) Late endosomes/lysosomes (green), microtubules (red), and nuclei (blue) were fluorescently visualized by specific antibodies or dyes (**g**), and distribution indices were quantified (**i**). Bar, 10 µm. The graph indicates the mean ± SEM of three independent experiments. * and **, $P < 0.05$ and $P < 0.005$ (two-tailed *t*-test, adjusted by the Holm-Sidak method), compared to si-NC samples, respectively.

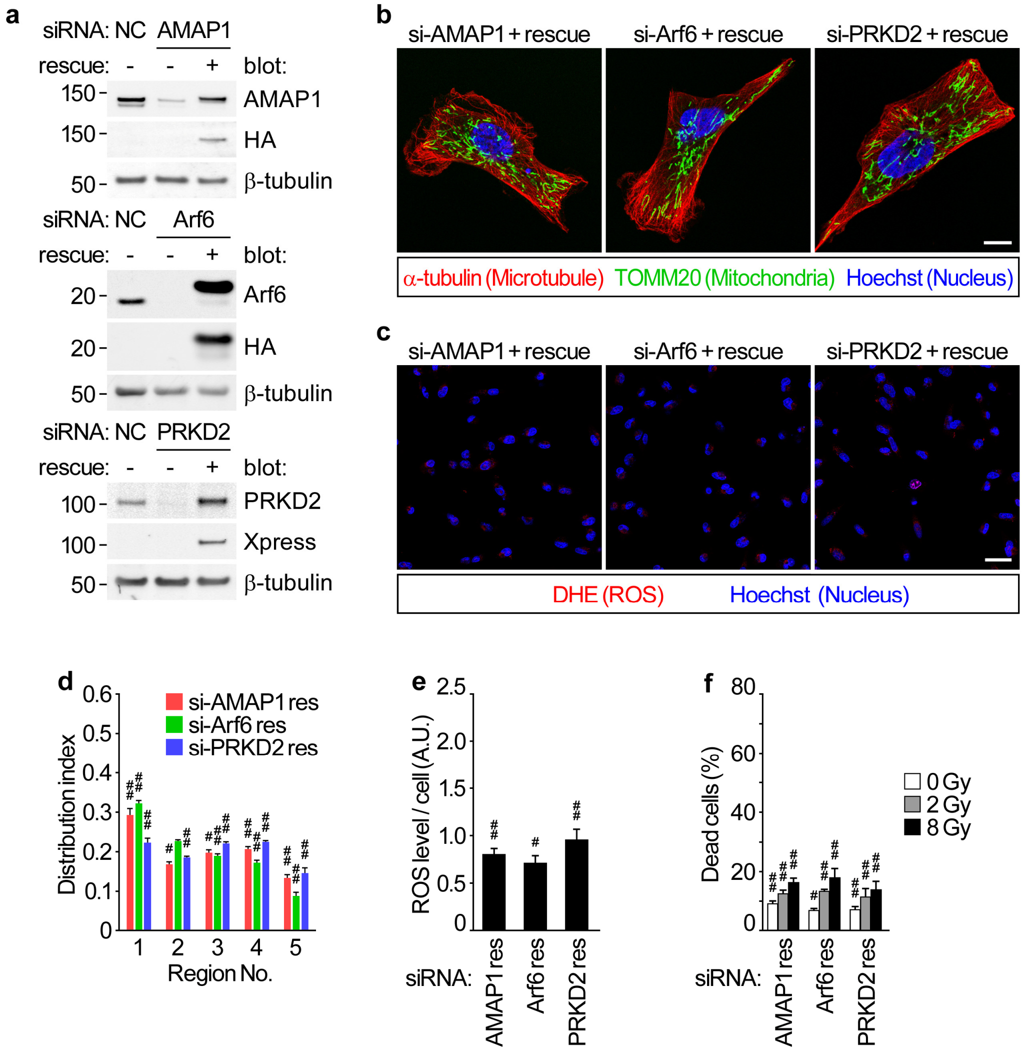

Supplementary Figure 2. Rescue of the phenotypes of siRNA treatments by expression of refractory cDNAs.

MDA-MB-231 cells, either parental or stably expressing cDNAs of AMAP1, Arf6, or PRKD2 refractory to #1 siRNA of each (indicated as rescue (-) and (+), respectively), were transfected with the indicated siRNAs. Protein expression of AMAP1, Arf6, or PRKD2 was analyzed by western blotting (**a**). Expression of refractory cDNAs was detected by the HA or Xpress tags that were fused to them. Mitochondria (green), microtubules (red), and nuclei (blue) were fluorescently visualized by specific antibodies or dyes. Bar, 10 μm (**b**). Mitochondrial distribution was quantified (**d**). ROS production was visualized by DHE (**c**), and quantified (**e**). Cumulative cell death after IR treatment was also measured (**f**). All graphs indicate the mean ± SEM of three independent experiments. #, $P < 0.05$; ##, $P < 0.005$ (two-tailed *t*-test, adjusted by the Holm-Sidak method). # and ##, comparison to the corresponding samples (i.e., si-AMAP1 alone vs. siAMAP1 + refractory cDNA) in Fig. 1 and 3.

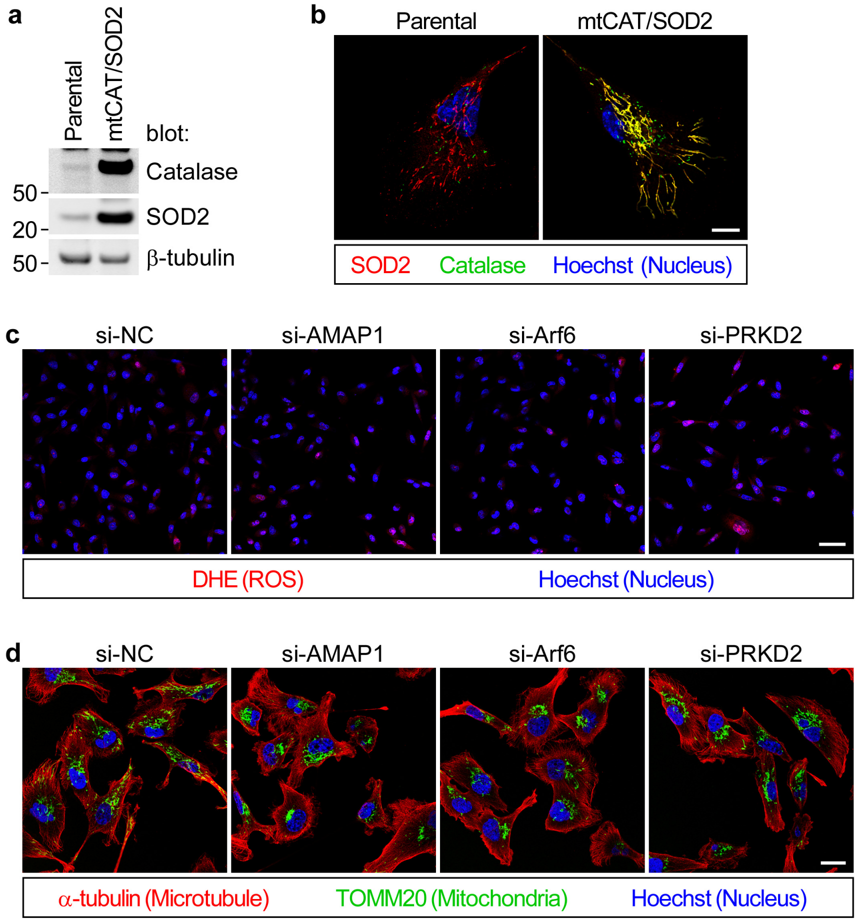

Supplementary Figure 3. Effects of the overexpression of mtCAT and SOD2.

MDA-MB-231 cells stably expressing mtCAT and SOD2 were transfected with siRNAs, as indicated. (**a,b**) Protein expression (**a**) and subcellular localization (**b**) of catalase (green) and SOD2 (red) in parental and mtCAT/SOD2-expressing cells. Bar, 10 µm. (**c**) ROS production visualized by DHE (red). Nucleus staining with Hoechst 33342 (blue) is also shown. Bar, 50 µm. (**d**) Immunostaining of TOMM20 (green) and α-tubulin (red) by specific antibodies. Nuclei were stained with Hoechst 33342 (blue). Bar, 20 µm.

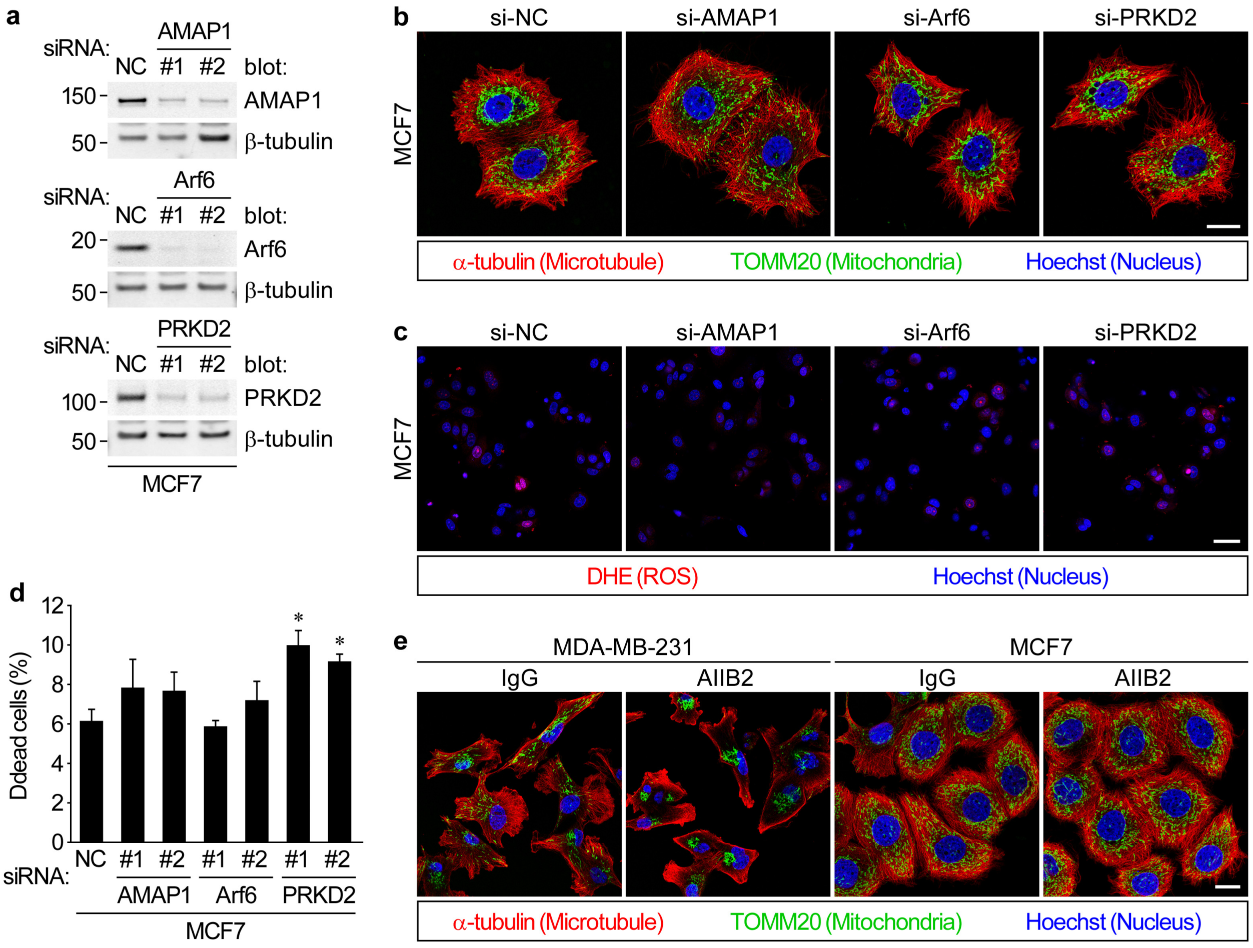

Supplementary Figure 4. Regulation of mitochondrial distribution and ROS generation by the Arf6-AMAP1-PRKD2-β1 integrin axis is context dependent.

(**a-d**) MCF7 cells were transfected with the indicated siRNAs. Protein expression of AMAP1, Arf6, or PRKD2 was analyzed by western blotting (**a**). Immunostaining of TOMM20 (green) and α-tubulin (red) by specific antibodies. Nuclei were stained with Hoechst 33342 (blue). Bar, 10 µm (**b**). ROS production visualized by DHE (red). Bar, 50 µm (**c**). Quantification of cumulative cell death after siRNA transfection (**d**). (E) MDA-MB-231 and MCF7 cells cultured on collagen I-coated plates were treated with an inhibitory antibody to β1 integrin (clone AIIB2). Cells were immunostained with TOMM20 (green) and α-tubulin (red) antibodies. Nuclei were also stained with Hoechst 33342 (blue). Bar, 20 µm. The graph indicates the mean ± SEM of three independent experiments. * indicates $P < 0.05$ (two-tailed *t*-test, adjusted by the Holm-Sidak method).

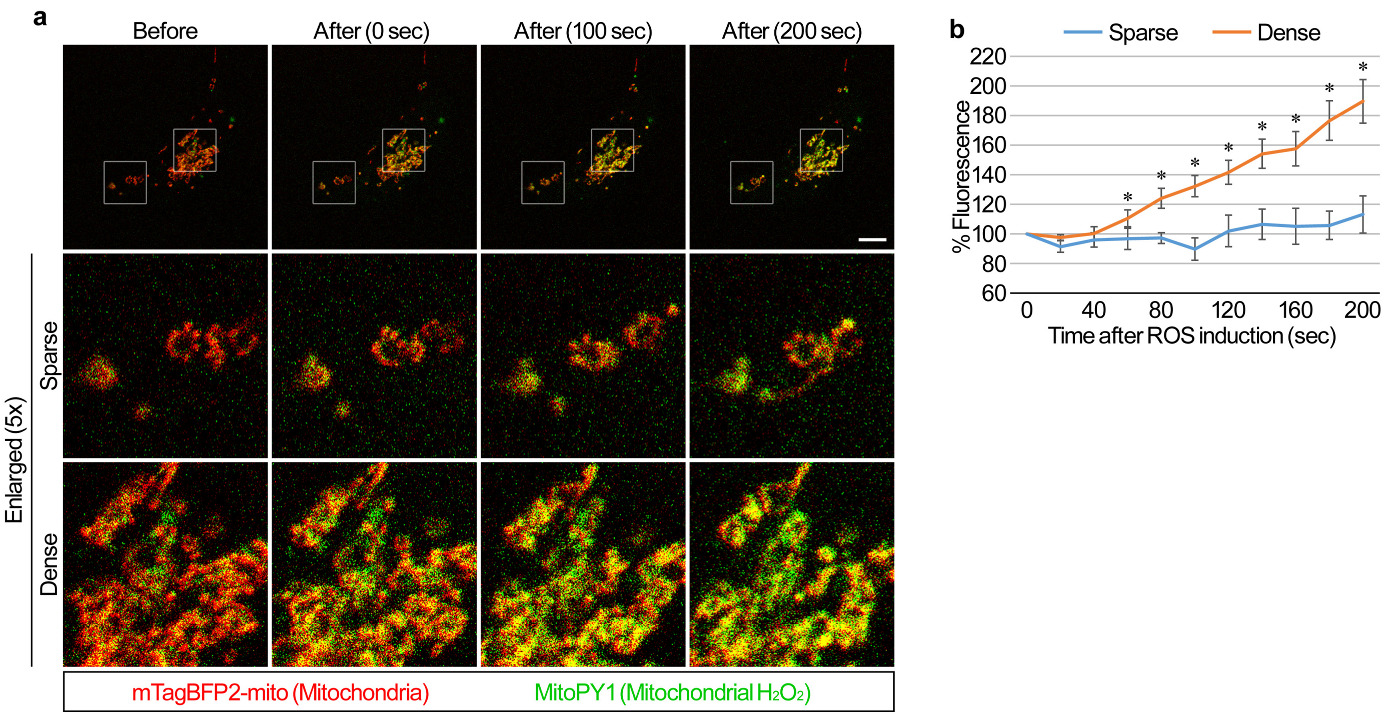

Supplementary Figure 5. Amplification of cytoplasmic ROS by dense mitochondria.

(**a**) Visualization of mitochondrial H_2O_2 in MDA-MB-231 cells stably expressing KillerRed-NES (not shown) and mTagBFP2-mito (pseudo-colored in red). H_2O_2 was visualized using MitoPY-1 (green). Time-lapse images before and after ROS induction by photoexcitation of KillerRed-NES, are shown. The white squares indicate the enlarged areas shown on the right. Bar, 10 μm. (**b**) Quantification of the time-dependent changes of mito-PY1 fluorescence in mitochondria-dense or -sparse areas in the same cell. The graph indicates the mean ± SEM of 10 independent experiments. * indicates $P < 0.05$ (two-tailed *t*-test, adjusted by the Holm-Sidak method).

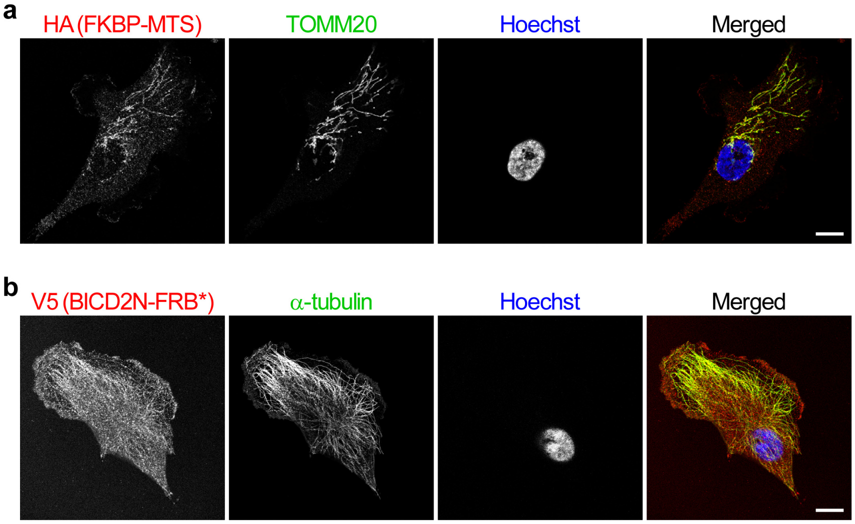

Supplementary Figure 6. Localization of components for the drug-regulatable mitochondrial trafficking system. MDA-MB-231 cells stably expressing FKBP-MTS and BICD2N-FRB*, tagged with HA and V5 epitopes, respectively, were immunostained as indicated. (**a**) Localization of FKBP-MTS was analyzed by immunostaining with HA (red) and TOMM20 (green) antibodies. (**b**) Localization of BICD2N-FRB* analyzed by immunostaining with V5 (red) and α-tubulin (green) antibodies. Nucleus staining with Hoechst 33342 (blue) is also shown. Bars, 10 µm.

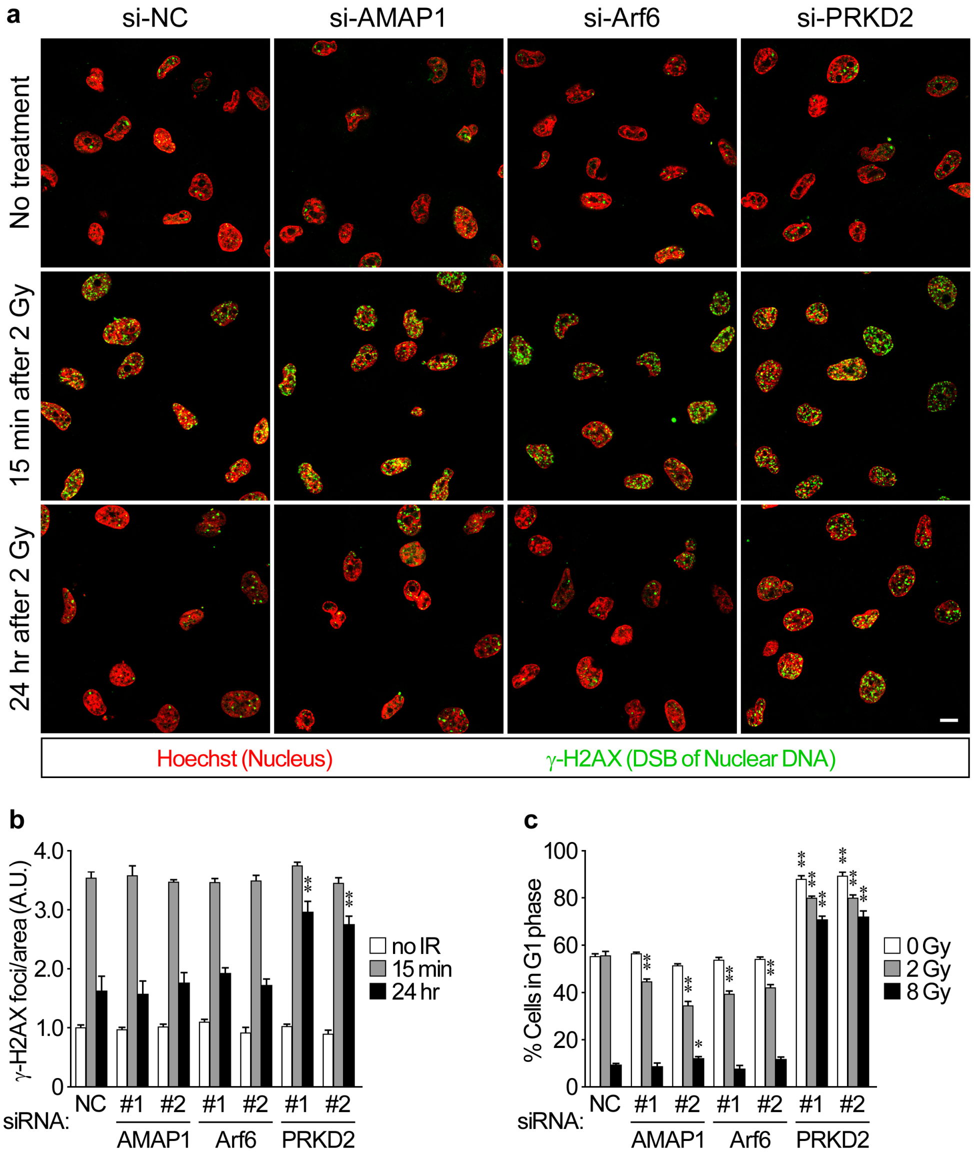

Supplementary Figure 7. Roles of PRKD2 in DSB repair and cell cycle regulation.

MDA-MB-231 cells transfected with the indicated siRNAs were exposed to the indicated doses of IR. (**a**,**b**) Cells were immunostained with the γ-H2AX antibody (**a**) at the indicated time points after 2 Gy IR exposure. Bar, 10 μm. Number of γ-H2AX foci per nuclear cross section was also calculated (**b**). (**c**) Percentage of cells in the G1 phase was analyzed using Fucci probes 24 hours after exposure to the indicated doses of IR. All graphs show the mean ± SEM of three independent experiments. * and ** indicate $P < 0.05$ and $P < 0.005$ (two-tailed *t*-test, adjusted by the Holm-Sidak method), compared to si-NC samples, respectively.

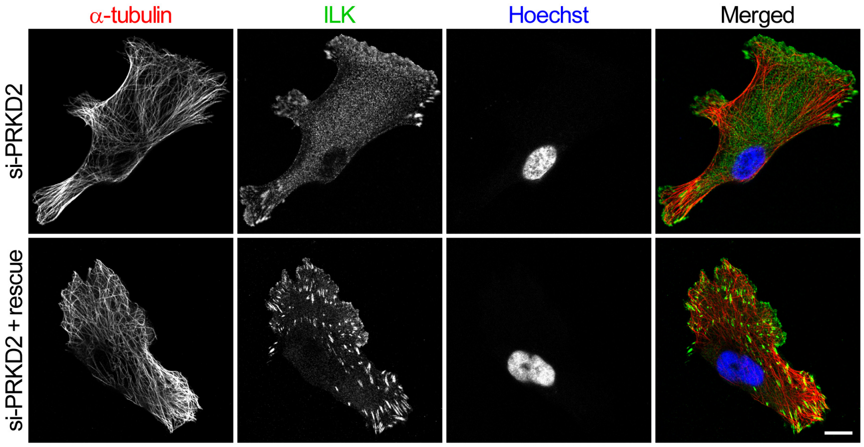

Supplementary Figure 8. Roles of PRKD2 in ILK localization.

MDA-MB-231 cells, either parental or stably expressing PRKD2 cDNA refractory to siRNA, were transfected with PRKD2 siRNA. Cells were immunostained with ILK (green) and α-tubulin (red) using their specific antibodies. Nuclei were stained with Hoechst 33342 (blue). Bar, 10 μm.

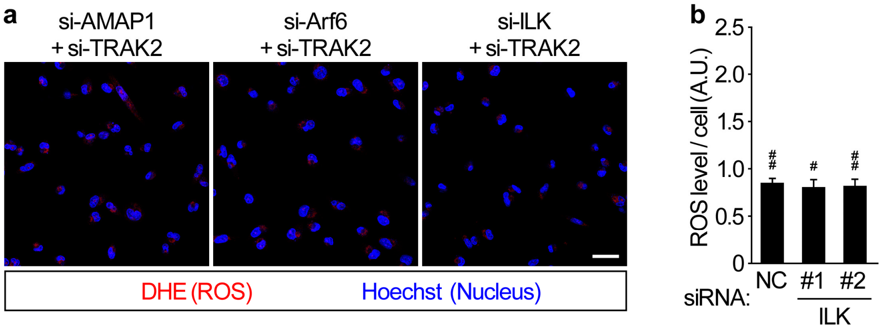

Supplementary Figure 9. Increase in ROS levels caused by inhibition of the Arf6-AMAP1-ILK axis can be rescued by knockdown of TRAK2.

MDA-MB-231 cells were transfected with the combinations of siRNAs, as indicated. (**a**) ROS production was visualized by DHE (red). Nucleus staining with Hoechst 33342 (blue) is also shown. Bar, 50 μm. (**b**) Quantification of DHE fluorescence. The graph indicates the mean ± SEM of three independent experiments. #, $P < 0.05$; ** and ##, $P < 0.005$ (two-tailed t-test, adjusted by the Holm-Sidak method). # and ##, comparison to the corresponding samples (i.e., si-AMAP1 alone vs. siAMAP1 + si-TRAK2) in Fig. 1.

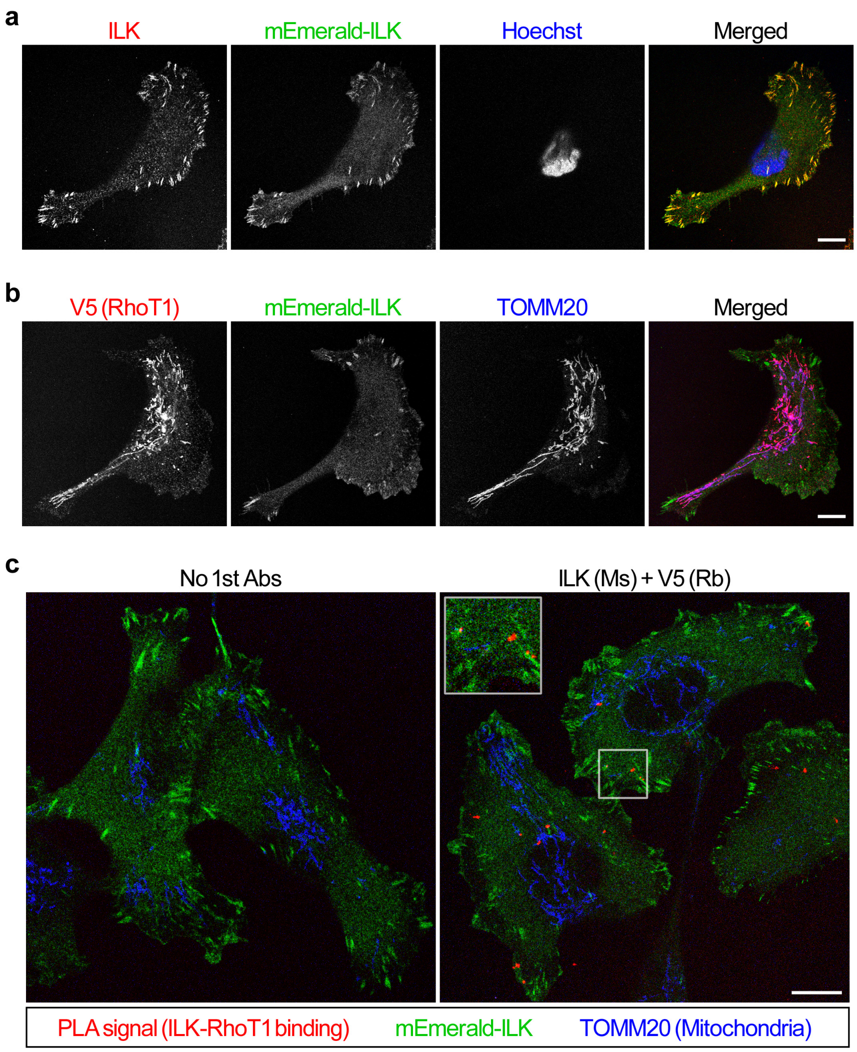

Supplementary Figure 10. Intracellular location of the ILK-RhoT1 interaction.

MDA-MB-231 cells were stably transfected with vectors encoding mEmerald-ILK and V5-RhoT1. (**a,b**) Cells were immunostained with an anti-ILK mouse antibody (red, **a**), or with an anti-V5 rabbit antibody (red, **b**). Nuclei and mitochondria were stained with Hoechst 33342 (blue, **a**) and the anti-TOMM20 antibody conjugated with Alexa 405 (blue, **b**), respectively. Fluorescence of mEmerald-ILK (green) is also shown. (**c**) PLA was performed using the anti-ILK and anti-V5 antibodies used in (**a**) and (**b**). PLA signal spots are shown in red, and mEmerald-ILK and mitochondria are shown in green and blue, respectively, as above. As a control, the PLA reaction was also performed without the primary antibodies (No 1st Abs). The white squares indicates the enlarged area. Bar, 10 µm.

Figure 1a

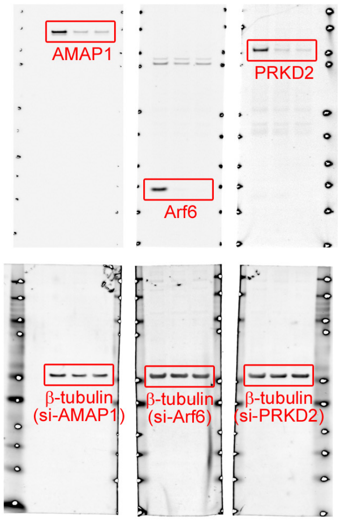

Figure 3f

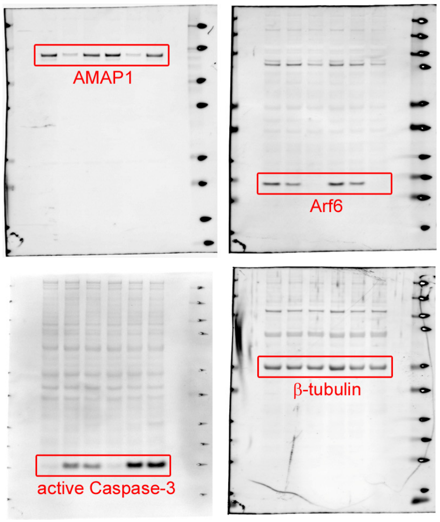

Figure 4a

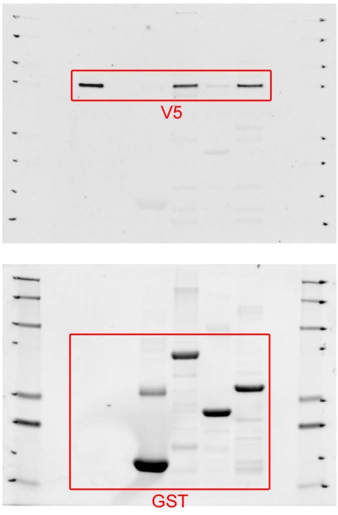

Figure 4b

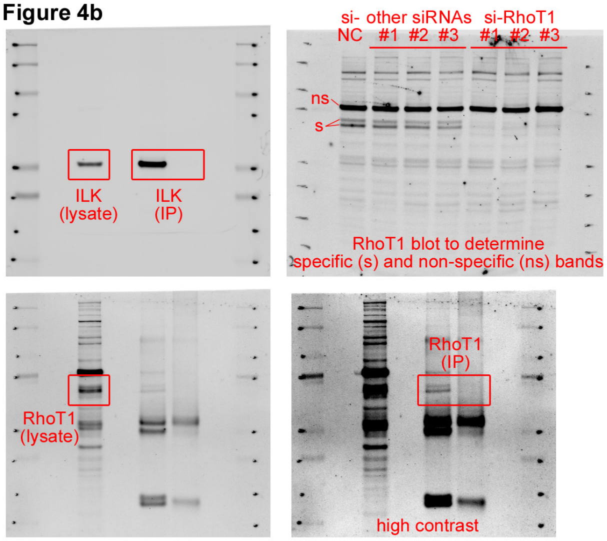

Figure 4c

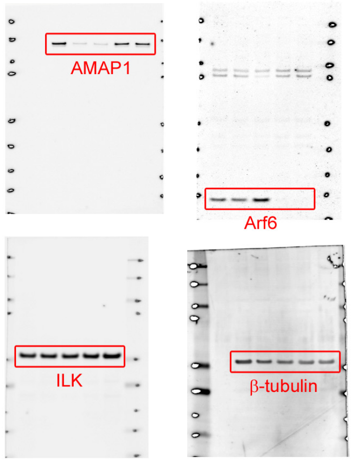

Figure 4e

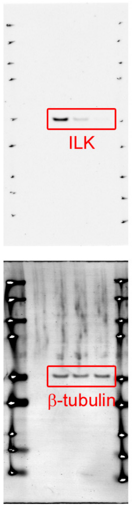

Figure 4f

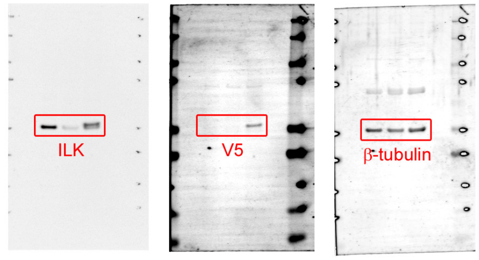

Figure 5a

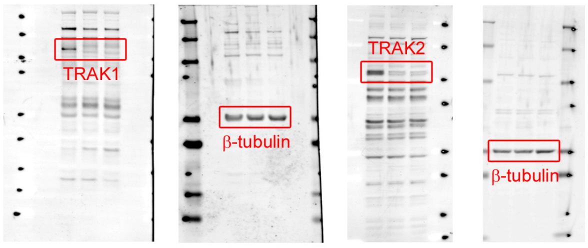

Figure 5d

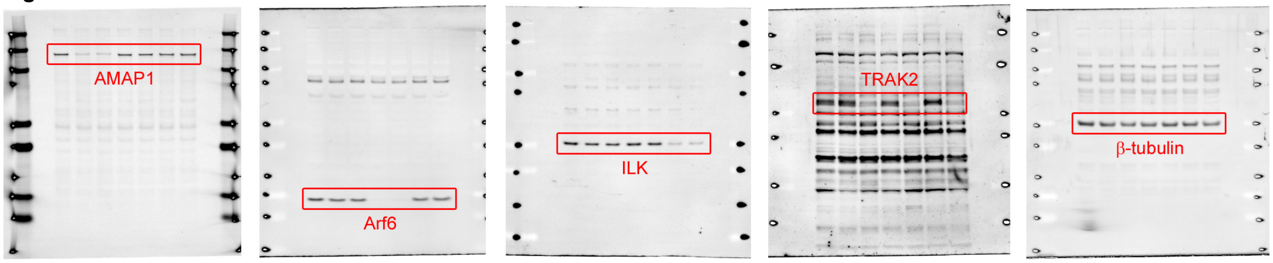

Figure 5g

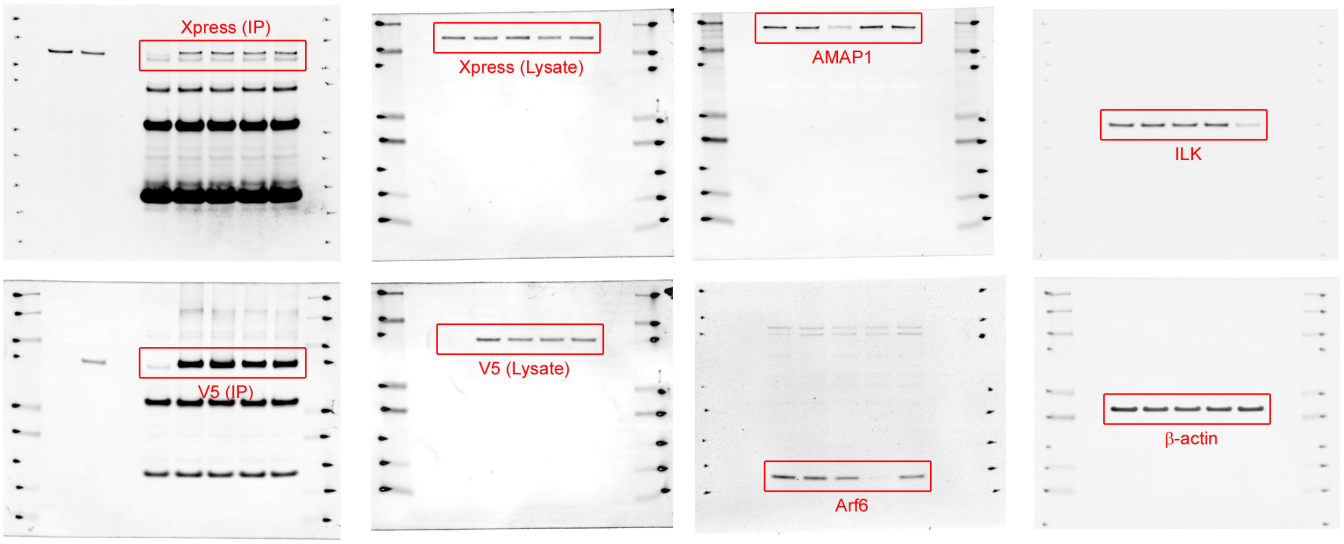

Figure 5h

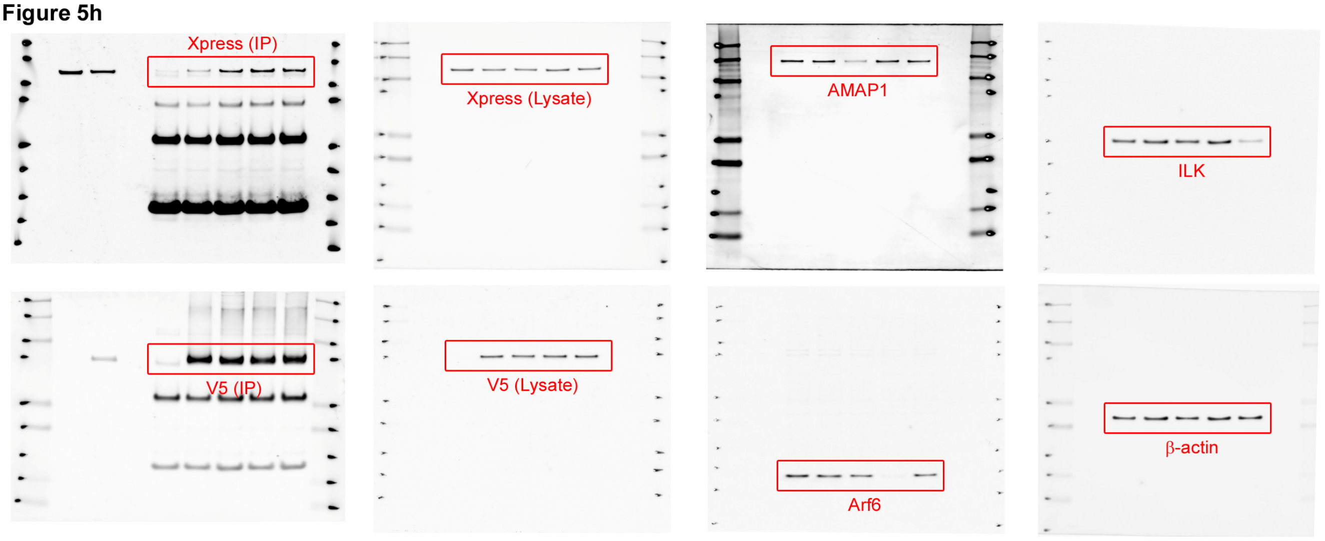

Figure 5i

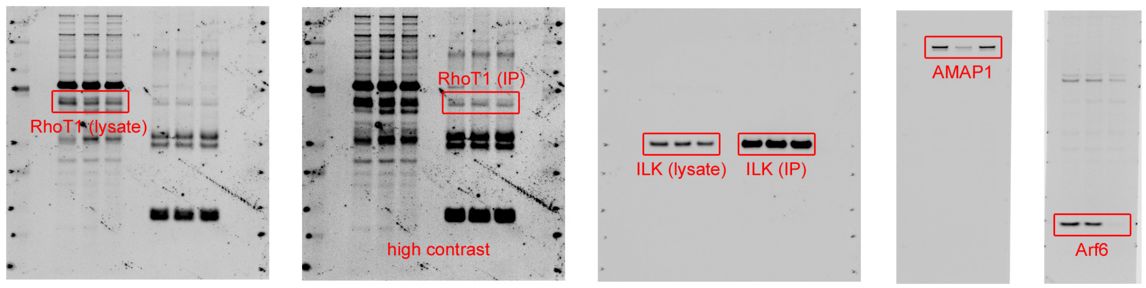

Figure 6a

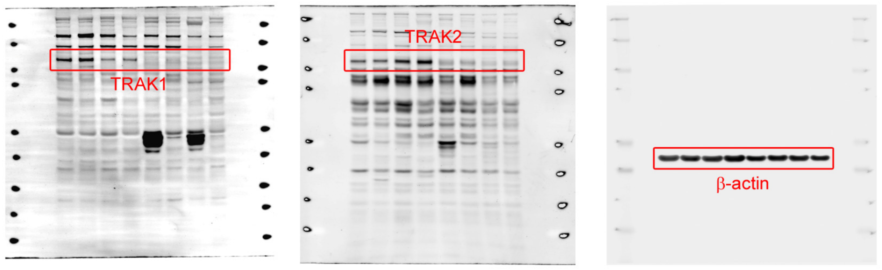

Figure 7b

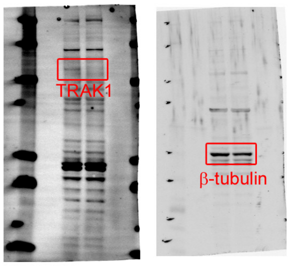

Figure 7d

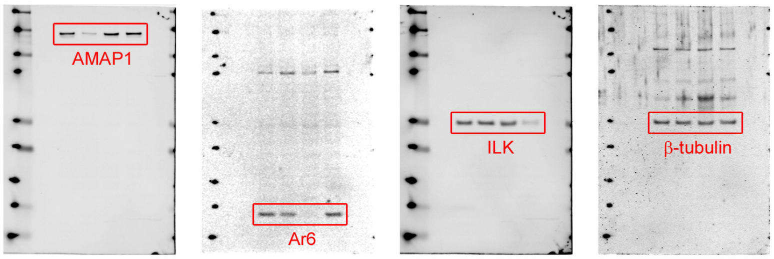

Figure 7e

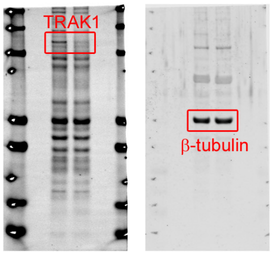

Supplementary Figure 2a

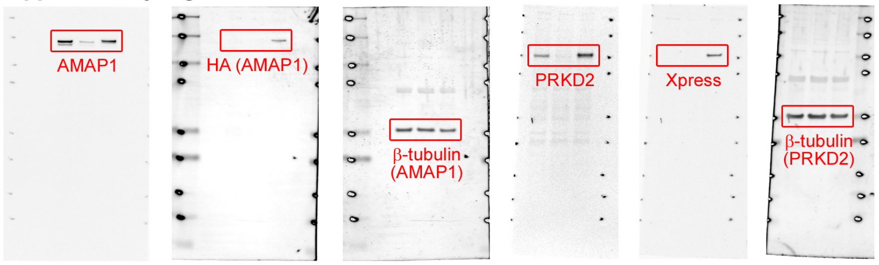

Supplementary Figure 3a

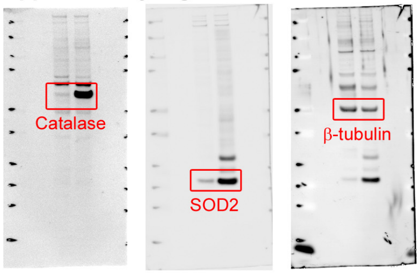

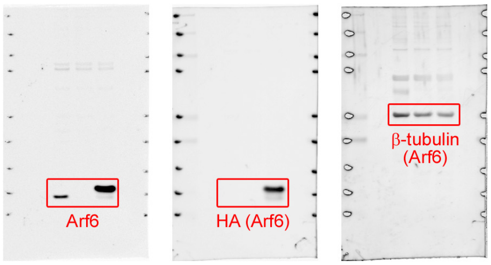

Supplementary Figure 4a

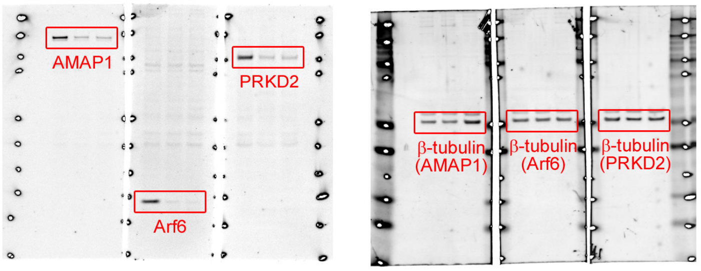

Supplementary Figure 11. Uncropped scans of western blots.
